# Hierarchical Multi-Species Modeling of Carnivore Responses to Hunting, Habitat and Prey in a West African Protected Area

**DOI:** 10.1371/journal.pone.0038007

**Published:** 2012-05-30

**Authors:** A. Cole Burton, Moses K. Sam, Cletus Balangtaa, Justin S. Brashares

**Affiliations:** 1 Department of Environmental Science, Policy and Management, University of California, Berkeley, California, United States of America; 2 Alberta Biodiversity Monitoring Institute, University of Alberta, Edmonton, Alberta, Canada; 3 Wildlife Division of the Forestry Commission of Ghana, Accra, Ghana; Australian Wildlife Conservancy, Australia

## Abstract

Protected areas (PAs) are a cornerstone of global efforts to shield wildlife from anthropogenic impacts, yet their effectiveness at protecting wide-ranging species prone to human conflict – notably mammalian carnivores – is increasingly in question. An understanding of carnivore responses to human-induced and natural changes in and around PAs is critical not only to the conservation of threatened carnivore populations, but also to the effective protection of ecosystems in which they play key functional roles. However, an important challenge to assessing carnivore communities is the often infrequent and imperfect nature of survey detections. We applied a novel hierarchical multi-species occupancy model that accounted for detectability and spatial autocorrelation to data from 224 camera trap stations (sampled between October 2006 and January 2009) in order to test hypotheses about extrinsic influences on carnivore community dynamics in a West African protected area (Mole National Park, Ghana). We developed spatially explicit indices of illegal hunting activity, law enforcement patrol effort, prey biomass, and habitat productivity across the park, and used a Bayesian model selection framework to identify predictors of site occurrence for individual species and the entire carnivore community. Contrary to our expectation, hunting pressure and edge proximity did not have consistent, negative effects on occurrence across the nine carnivore species detected. Occurrence patterns for most species were positively associated with small prey biomass, and several species had either positive or negative associations with riverine forest (but not with other habitat descriptors). Influences of sampling design on carnivore detectability were also identified and addressed within our modeling framework (e.g., road and observer effects), and the multi-species approach facilitated inference on even the rarest carnivore species in the park. Our study provides insight for the conservation of these regionally significant carnivore populations, and our approach is broadly applicable to the robust assessment of communities of rare and elusive species subject to environmental change.

## Introduction

Protected areas are a cornerstone of global conservation efforts to shield wildlife from anthropogenic impacts such as excessive hunting and habitat loss [1], [2]. The number and extent of protected areas (hereafter PAs or parks) have grown exponentially over recent decades, yet their ecological effectiveness is increasingly in question since many are small and isolated, lack adequate capacity for law enforcement, and are beset by illegal hunting and resource collection [3]–[6]. Moreover, rapid human population growth around PAs and the attractiveness of a park's otherwise scarce resources may result in elevated impacts at PA edges and cause increased isolation and edge effects [7], [8]. Such detrimental effects may be particularly severe for large, wide-ranging species prone to conflict with humans, most notably mammalian carnivores [9]–[11]. Effective PA networks are presumed to be key to the long-term viability of many carnivore species [12], [13], so an understanding of carnivore responses to human-induced and natural changes in and around PAs is critical not only to the conservation of threatened carnivore populations, but also to the protection of ecosystems in which they play important functional roles [14], [15].

Anthropogenic activities can impact carnivore populations directly and indirectly. Direct persecution is often a major threat to both large- and smaller-bodied carnivores as they may be hunted as trophies [16], for traditional uses like bushmeat [17], [18], and in retaliation for real or perceived threats to livestock or human life [19], [20]. Furthermore, many carnivore species are wary by nature and avoid areas of elevated human activity, such that even non-lethal activities (e.g. pastoralism, tourism) can influence their occurrence and viability [21], [22]. Besides these direct anthropogenic influences, hunting of prey populations can be an important indirect human impact on carnivore viability, given that the availability of suitable prey is a key determinant of carnivore occurrence and abundance [23]–[25]. Finally, habitat destruction can influence carnivores both directly and indirectly and is predicted to affect some species more than others (e.g., [26]). Effects of habitat change on carnivores may be mediated through the response of their prey, or other factors such as associated changes in disease dynamics [27].

Management efforts attempt to address the threats faced by carnivores in and around PAs through more effective enforcement of anti-poaching laws [28], [29], creation of partially protected buffer zones or corridors [11], [30], [31], restoration of habitat and prey (i.e., increasing predator carrying capacity; [25]), resolution of human-carnivore conflict [32]–[35], and metapopulation management to minimize loss of genetic diversity [31], [36]. However, in practice, these interventions are exceedingly difficult to implement for political, economic and social reasons. Given limited resources, PA managers must identify approaches that will provide the greatest conservation return on their investment, but designing and implementing these optimal strategies requires an understanding of carnivore responses to specific stressors. Furthermore, management actions targeted to address responses of entire carnivore communities may be more ecologically- and cost-effective than single-species approaches [37]–[41], which have typically focused only on larger-bodied carnivores.

A significant challenge to assessing carnivore communities and their responses to anthropogenic impact is the often infrequent and imperfect nature of survey detections [42]. Accurate modeling of species' distributions and habitat suitability typically requires a large number of observations and implicitly assumes that species are absent from surveyed locations where they are not detected [43]. However, the rare and elusive nature of many carnivore species frequently translates into small sample sizes and low detection probabilities, and hence biased population estimates [44]. Fortunately, recent advances in survey and statistical techniques can be applied to address this challenge. Camera trapping has proven an effective technique for detecting cryptic carnivores [45], [46], particularly for mark-recapture estimation of abundance for individually identifiable species [47], [48]. The nature of camera-trap surveys – with camera stations sampling continuously over time at specific sites – is well-suited to an occupancy modeling analytical framework that explicitly accounts for imperfect detection [41], [49]. The use of occupancy as a surrogate for abundance has been widely adopted [50] and is appropriate for widespread, low-density carnivore populations [51]. Furthermore, recently developed hierarchical multi-species occupancy models capitalize on the information content of multiple detection histories to improve inference for rare species and generate insight on aggregated responses of wildlife communities [52], [53]. Hierarchical models also provide a flexible modeling framework capable of addressing other important assumptions, including spatial independence among sampling sites [54], [55]. Models explicitly accounting for spatial autocorrelation are increasingly being applied to the estimation of animal occurrence patterns [56]–[58], and have in many cases been shown to improve inference [59], [60].

In this study, we developed a Bayesian hierarchical multi-species occupancy model accounting for spatial autocorrelation to assess patterns of carnivore occurrence in relation to key landscape features in Mole National Park, Ghana (hereafter MNP). MNP is among the largest protected areas in West Africa and, as with most of this region, its carnivore populations are poorly studied yet subjected to considerable pressure from the region's high human densities and widespread hunting for bushmeat [61], [62]. Illegal hunting is a central management concern in MNP, and previous work indicates that the park's carnivore community has been heavily impacted, with evidence of human-caused mortality and the decline and likely extirpation of several species [18]. Nevertheless, the direct and indirect effects of hunting on MNP's carnivore populations are unknown. We used law enforcement patrol records to develop a spatially explicit index of hunting pressure and test the hypothesis that hunting is a major determinant of carnivore occurrence patterns in MNP. Using a Bayesian model selection framework, we further tested the importance of other anthropogenic and natural factors on carnivore occurrence, including prey availability, habitat type, and law enforcement protection. Our approach not only informs the conservation of MNP's regionally important carnivore populations, but is also broadly applicable to the robust assessment of rare and elusive species subject to environmental change.

## Methods

### Ethics statement

We thank the Wildlife Division of the Forestry Commission of Ghana for their permission to conduct this work in Mole National Park (Research project permit code 01/09/2006).

### Study area

MNP is the largest of Ghana's protected areas and covers approximately 4600 km^2^ of woodland savanna habitat in the country's Northern Region (∼09^o^11′–10^o^06 N and 01^o^22′–02^o^ 16′ W). Elevation ranges from 120–490 m and open savanna woodland is the dominant habitat type, with tree cover averaging about 30% and grasses reaching 2–3 m in height during the April-to-October wet season [63]. Mean annual rainfall is approximately 1100 mm and most of the park's rivers are seasonal, draining into the White Volta River.

### Camera trap survey

**Figure 1:**
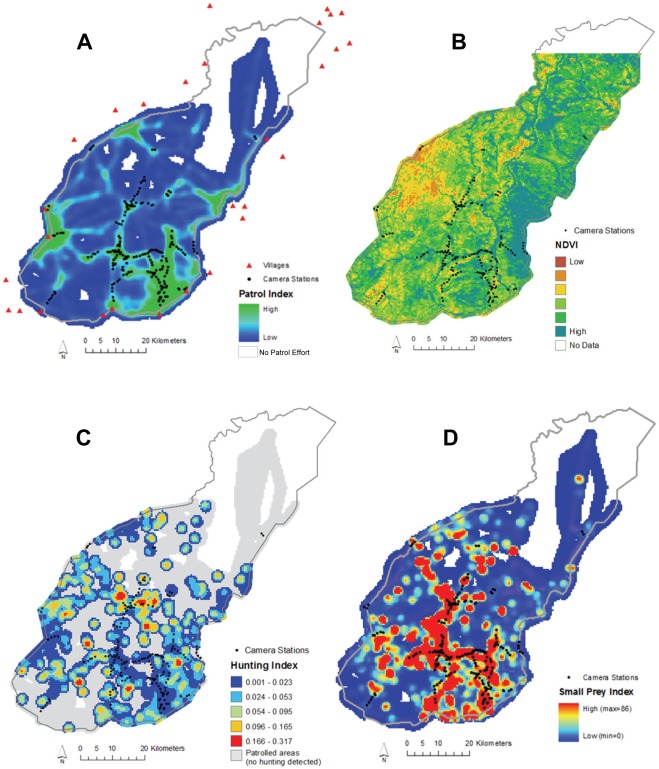
Camera-trap locations (**n = 224**) **and indices of patrol effort, hunting activity, habitat, and prey biomass in Mole National Park, Ghana.** (A) Index of law enforcement patrol effort (i.e., “protection”) calculated as the density of patrol pathways covered between Oct. 2006 and May 2008 (also showing the location of villages within 10 km of the park boundary); (B) NDVI, the normalized difference vegetation index (from MODIS/Terra sensor) summed over the study period (i.e., integrated NDVI, Oct. 2006 – Jan. 2009); (C) Index of illegal hunting activity detected by law enforcement patrols (observations per unit patrol effort); (D) Patrol-based, multi-season index of biomass for prey species weighing less than 18 kg (standardized by patrol effort). No data were obtained in the white areas within the park boundary.

We conducted a camera trap survey between October 2006 and January 2009 to estimate carnivore occurrence patterns in MNP (see also [18], [64]). For this study, we used data from 224 camera stations deployed along gradients of proximity to park boundary, potential prey abundance, and availability of water and associated riparian forest habitat (Fig. 1). Our survey design was constrained by access limitations (particularly in northern portions of the park) and the number of available cameras, but we covered representative gradients using systematic sampling within 31 camera arrays targeting different portions of the park and different seasons (mean = 7.2 stations per array). Within an array, stations were spaced at about 1-km intervals near specific features expected to maximize carnivore capture probability, such as dirt roads, wildlife trails, waterholes, and salt licks (with a mix of stations on and off roads – see below). Most stations consisted of a single passive infra-red DeerCam DC-300 film camera trap unit (Non Typical, Park Falls, WI, USA) set on a tree at a height of about 40 cm, facing perpendicular to the expected direction of animal travel and approximately 3 m from the anticipated site of capture. Sampling effort at a station was calculated as the number of days for which a camera was set (or until the last photo was taken if the roll was fully exposed before collection) and total effort across the 224 stations was 4,867 trap-days (mean = 21.7, SD = 13.0, range = 3–93). Effort was concentrated in the central and southeastern portions of the park (Fig. 1) and during dry season months when access was greatest (∼70% of trap-days between October and April). Detection or non-detection of carnivore species was recorded at each station for each trap day, yielding a response variable representing an uncorrected or “naïve” estimate of carnivore occurrence across the sampling sites [50].

### Hypothesized predictors of carnivore occurrence

We hypothesized that spatial patterns of carnivore occurrence in MNP would be influenced by variation in hunting pressure and human disturbance, anti-poaching patrol effort, prey biomass and habitat type (Table 1). To test our hypotheses, we created spatially explicit indices representing each of these factors and extracted values for each sampling location (i.e., camera station) from the camera trap survey. Our intent was to examine general, management-relevant indices of expected importance across the carnivore community, given the lack of previous study and detailed species-specific knowledge for MNP. Analyses were conducted using ArcMap 9.3.1 (ESRI, CA, USA) and density surfaces (described below) were created using a kernel density estimator in the Spatial Analyst ArcMap extension (with a 2 km search radius and output resolution of 500×500 m).

**Table 1:** Factors hypothesized to influence patterns of carnivore occurrence in Mole National Park (MNP), with the corresponding index used, predicted direction of effect (i.e., negative or positive influence on occurrence, or both), source of data, and range of values across sampled sites.

Factor	Index	Predicted effect	Source	Range of values^a^
Hunting pressure	Relative frequency of poaching observations	−	MNP patrol system	0−0.20 obs./unit patrol effort
Human disturbance	Distance from park edge	−	MNP GIS data layer	0–22.4 km
Patrol protection	Relative anti-poaching patrol effort	+	MNP patrol system	1.3–245.5 units of patrol effort
Prey biomass^b^	Relative biomass of potential prey	+	MNP patrol system (multi-season) and camera trap detections (seasonal).	0–1722.1 kg/ unit patrol effort; 0–781.4 kg/trap-day
Small prey biomass^b^	Relative biomass of smaller prey (< 18kg)	+	MNP patrol system (multi-season) and camera trap detections (seasonal)	0–41.0 kg/unit patrol effort; 0–69.0 kg/trap-day
Riverine forest	Distance from nearest corridor of riverine forest	+/−	GIS data layer derived from Landsat image (GWD 2005)	0.01–7.2 km
Vegetation productivity	NDVI^c^	+/−	MODIS/Terra (MOD13Q1, 250m, lpdaac.usgs.gov)	1882–7720 (seasonal)^d^ 230,608–322, 297 (integrated)^d^

a Range of values for sampled camera stations. Data were normalized and standardized prior to analysis.

b Prey species are listed in Table S1. Species average adult body masses were taken from Jones et al. (2009). Total prey biomass was expected to have a greater influence on larger carnivores given the relative dominance of larger prey species. See Methods for details on the calculation of different indices from patrol and camera-trap data.

c NDVI = Normalized Difference Vegetation Index.

d See Methods for details on the seasonal and integrated measures of NDVI.

#### (i) Hunting pressure and human disturbance

Carnivore species are often killed in Ghana and elsewhere as a perceived threat to livestock and human life or for traditional purposes [18]. We therefore hypothesized that carnivores would be less likely to occur in portions of MNP experiencing heavy hunting pressure. We used spatially explicit observations of illegal hunting activity in the park made during law enforcement patrols [65] to construct an index of hunting pressure (there is no legal hunting within MNP). Evidence of illegal hunting – ranging from direct sightings and arrests to indirect signs such as hunting camps, traps or hunter footprints – were recorded by teams of 3–6 staff during frequent foot patrols across much of the park (Fig. 1; [64]), with specific locations determined using handheld GPS units. We used data from nearly 1,400 patrols conducted between October 2006 and May 2008 and comprising 688 observations of illegal hunting to create a density surface of hunting activity across the park. We then divided this by a similar density surface describing patrol effort (see below) to derive a spatial index of relative hunting pressure across the park (equivalent to a catch-per-unit-effort or CPUE index; cf. [65]). As an alternative measure of human disturbance in MNP, we calculated the Euclidean distance from each sampling location to the nearest boundary of the park. This simple index represents potential edge effect and is often used as a proxy for hunting pressure (and was highly correlated with distance to the nearest village, Pearson *r* = 0.91; Fig. 1).

#### (ii) Law enforcement protection

Law enforcement (“anti-poaching”) patrols are intended to deter illegal hunting activity and thereby provide protection to park wildlife [29], [65]. We hypothesized that carnivores would be more likely to occur in areas within MNP that were more effectively protected by a greater level of patrol effort. We anticipated that this effect might be distinct from that associated with the amount of hunting activity detected per unit patrol effort (above), given that hunters could have been avoiding more heavily patrolled areas and that patrol routes were influenced by many factors (e.g., access, wildlife abundance, management zones). Patrol teams recorded their locations with handheld GPS units at regular intervals along patrol routes, and we used pathways (i.e., joined locations) from the ∼1,400 patrols to construct a density surface of patrol effort across the park (Fig. 1).

#### (iii) Prey

The availability of suitable prey species is a key determinant of the distribution and abundance of carnivore populations (e.g., [23]). Prey availability may represent a natural influence on carnivores but could also reflect an indirect anthropogenic effect if prey are depleted by exploitation [25]. We used two data sources to create indices characterizing longer- and shorter-term spatial patterns of prey biomass in MNP. First, we used spatially referenced sightings of potential mammalian prey species recorded during the October 2006–May 2008 law enforcement patrols to create a kernel density surface representing the longer-term (i.e., multi-season) distribution of prey biomass. This dataset included approximately 8,600 sightings of nearly 58,000 individuals of 14 ungulate or primate species (median body mass = 30.5 kg, range = 3.7–592.7 kg; Table S1; see also [64]). Prey counts were converted to biomass estimates by multiplying the number of individuals of a particular species counted by the body mass of that species, using values of estimated average adult body mass from the PanTHERIA database [66]. The prey biomass surface was then divided by the patrol effort surface (as for hunting above) to create a spatial CPUE index of prey biomass. Since most of the carnivore species detected in MNP were of medium or small size (i.e., <15 kg; see Results and Table S1), we also calculated an index of small prey biomass including only the 7 species weighing less than 18 kg. This represented a relevant break point in the distribution of prey body masses between larger ungulates and smaller species (Table S1), and indices calculated using finer body mass subdivisions were correlated with the broader indices, thus we considered it a useful compromise for characterizing prey availability across the diverse carnivore community (although we note that the patrol-based indices do not include prey items suitable for small carnivores; see below).

Our second prey index was derived from the camera trap survey and represented an estimate of prey biomass at each camera site for the specific period over which it was sampled (i.e., short term, seasonally specific). The number of detections of a particular prey species at a given camera station (excluding multiple photos of ostensibly the same individual obtained <5 minutes apart) was multiplied by that species' average adult body mass (obtained from [66] for mammals and [67] for birds) and standardized by sampling effort into a CPUE index of kg of prey biomass per 100 camera trap days. Twenty-seven potential prey species were detected during the camera survey, including 20 mammal and 7 bird species (median body mass = 8.0 kg, range = 0.1–592.7; Table S1), and, as for the patrol sightings, separate biomass indices were calculated for all prey species combined and for the 20 smaller prey species weighing less than 18 kg. Camera trap detections included many more small prey species than patrol observations, and thus likely translated into more relevant indices for smaller carnivore species, although all of our prey indices omit or underrepresent the smallest prey items (e.g., small rodents, insects) and are therefore less directly suitable for the smallest carnivores (e.g., mongooses, genet). In the absence of additional data, we made the assumption that variation in biomass of the smallest prey would be indirectly reflected in the indices of larger prey and/or habitat (see below), but we suggest that future work could test this assumption.

#### (iv) Habitat

MNP's habitat is dominated by relatively intact open woodland savanna and we hypothesized that habitat heterogeneity would have a less pronounced effect on carnivore occurrence patterns than variation in hunting pressure or prey biomass. Nevertheless, the park experiences pronounced seasonal variation in vegetative cover – with dense grasses growing 2–3 m high in the wet season and frequently burned in the dry season – and narrow bands of riverine forest represent distinctive habitat features associated with important water sources. We therefore calculated three habitat indices, with the first being simply the Euclidean distance from each sampling site to the nearest band of riverine forest (demarcated from a Landsat-derived GIS map layer provided by park management, [63]). Our second and third habitat indices were based on the normalized difference vegetation index (NDVI), a measure of vegetation productivity [68], [69] that has been linked to occurrence patterns for many wildlife species [70], [71], including carnivores [37]. We used the NDVI derived from the MODIS sensor (Global MOD13Q1 product from the Terra satellite, 16-day composite image at 250 m resolution, downloaded from http://lpdaac.usgs.gov) to calculate both seasonally specific and longer-term measures of vegetation biomass in MNP. The former captured seasonal variation and corresponded to the 16-day composite NDVI value most closely matched to the period over which a given camera station was sampled (using the average of multiple composite values for stations sampled for more than 16 days or over a period split across two or more composite time frames). Our longer-term or “integrated” measure represented more stable spatial variation in vegetation biomass (i.e., different habitat types) and consisted of the sum of all 16-day composite NDVI values at a sampling location over the entire period of our survey (Oct. 2006 – Jan. 2009; [69]).

We did not explicitly test the effect of intraguild interactions on carnivore occurrence patterns, though we note its potential importance [72] and suggest it as a factor for future investigation (e.g., [73], [74]).

### Covariates of carnivore detectability

Our modeling framework for estimating carnivore occurrence patterns (described below) explicitly accounts for heterogeneity in carnivore detection probability. In addition to species-level heterogeneity, we anticipated that several site-level factors may have affected the probability of detecting a carnivore species (given its occurrence), so we included them as covariates in our model-based hypotheses to control for such “nuisance” effects on the estimation of occurrence probability. Firstly, we hypothesized that heavy hunting pressure and human disturbance may not only decrease the probability of carnivore occurrence, but could also make carnivores wary and thus more difficult to detect where they do occur. We therefore included the indices of relative hunting pressure and distance from park edge (see above) as covariates on detection as well as occurrence. We further hypothesized that certain aspects of our sampling design could have introduced spatial heterogeneity in detectability. Many of our camera stations were set on dirt roads or tracks (n = 90), which could have been used or avoided by certain species more often than adjacent areas lacking such features. We therefore tested for such an effect of roads by including a binary covariate on detection indicating whether or not a station was set along a park road. A small subset of camera stations (n = 17) consisted of a paired set of two camera units rather than the typical single unit (as part of a concurrent study on density estimation), raising the possibility that such paired stations had higher detection probabilities, so we included another indicator covariate distinguishing them from single-camera stations. While most of our stations were set by one field team led by A.C.B. for consistency, a portion was established by a second field team (n = 65), potentially introducing variation in detectability due to differences in set technique, so we included a third binary covariate indexing the set team. Finally, to account for marked variation between wet and dry seasons in factors that could affect camera performance at a site – such as ambient temperature or density of background vegetation – we included a fourth binary covariate on detection indexing the season in which a station was sampled (“dry” = median sampling date within October-April, “wet” = median date within May–September).

Prior to analysis, all continuous variables were examined for outliers, normalized with a fourth-root transformation (except for edge and the two NDVI variables; other variables were right-skewed prior to transformation), and standardized to have mean zero and unit variance (to improve convergence of model estimates and facilitate interpretation of relative effect sizes; [75], [76]). We tested variables for collinearity using correlation coefficients (Spearman *r_s_* for all variables and Pearson *r* for normalized continuous variables) and the variance inflation factor [77], [78]. All statistical tests were performed in program R version 2.11.1 [79]. Our hypothesized covariates of carnivore occurrence and detection probabilities were not strongly collinear (| *r_s_* |<0.57, | *r* |<0.65, variance inflation factor <3.3; Table S2), suggesting that they represented different attributes of the MNP environment (e.g., variation in seasonal vs. long-term prey or vegetation biomass).

### Background on modeling framework

We applied a multi-species occupancy modeling framework [54] to carnivore detection data from our camera trap survey. This framework represents a hierarchical formulation and extension of the single-species occupancy modeling approach described by MacKenzie et al. [80], and is essentially a robust adaptation of the logistic regression model frequently applied to species “presence-absence” data [43], [50]. A key advantage of the occupancy modeling approach is the explicit estimation of detection probability, providing a means to overcome the problematic assumption of perfect detection (i.e., species always being detected where they occur). The general framework requires repeated sampling of a site over a period considered closed to changes in occupancy status, and uses this temporal replication to estimate the probability that a species not detected at a site could have in fact been present (i.e., false absence). We treated consecutive trap days as repeat surveys at a given camera station and considered the occurrence of a species at a station equivalent to its use of the habitat at that site during the sampling period (assuming random species movement relative to a site rather than considering sites to be permanently “occupied”, and accordingly that detection probability includes availability for detection; [50]: 105). We also treated our entire survey period as one “season” in that most sites were not re-sampled across seasons, the carnivore community was assumed to be closed (i.e., no species extinctions or colonizations), and we did not wish to estimate site-specific probabilities of extinction or colonization over time (cf. [81]).

The multi-species model extends the single-species approach by capitalizing on additional information contained in multiple species' detection histories across a sampled community, simultaneously estimating occurrence and detection probabilities for all species. It assumes that an individual species' response comes from a common community-level distribution of responses. Species-specific parameters are thus treated as random effects governed by an associated community-level “hyper-parameter” (i.e., the hierarchical component). In this way, collective data on the entire carnivore community can improve species-specific estimates of occurrence, even for those species rarely observed and for which a single-species approach would likely yield unreliable results [52]. This approach also facilitates robust inference on the aggregate response of an entire community [40], [53].

### Model structure

Our model assumes that site-specific occurrence for species *i* = 1,2, …,*N* at site *j* = 1,2,…,*J*, is an imperfectly observed (latent) random variable, *z*(*i*,*j*), which is the outcome of a Bernouilli trial, *z*(*i*,*j*) ∼ Bern(*ψ_ij_*), where *ψ_ij_* is the probability that species *i* occurs at site *j*, and *z*(*i*,*j*) = 1 if it does occur and zero if it does not. Our observation data, *y*(*i*,*j*), representing the detection or non-detection of species *i* at site *j* during the camera trap survey, are conditional upon the true occurrence state, *z*(*i*,*j*), and are also assumed to be Bernouilli random variables if species *i* is present (that is if *z*(*i*,*j*) = 1) and are fixed zeros if species *i* is absent (i.e., if *z*(*i*,*j*) = 0, then *y*(*i*,*j*) = 0 with probability 1). This observation model is specified as *y*(*i*,*j*) ∼ Bern(*p_ij_* ·*z*(*i*,*j*)) for *k_j_* independent trials, where *p_ij_* is the probability of detecting species *i* at site *j* if it is present, and *k_j_* is the number of trap days for which the camera station at site *j* was active. We assumed that all species present in the MNP carnivore community were detected at least once during the survey, and we therefore did not estimate the probability of there being additional species that went completely undetected. Previous work suggests a low probability of additional carnivore species occurring in the park [18], and we focused our attention on confirmed species toward which management attention could be directed (cf. [40], [53]).

As noted above, we hypothesized that occurrence and detection probabilities would vary by species and be affected by anthropogenic and natural features of the park (as well as effects of sampling on detection). We incorporated these effects into the model linearly using the logit link function, with the general form of logit(*ψ_ij_*) =  *φ_i_*+*α_j_* and logit(*p_ij_*) = *ηi*+*β_j_*, where *φ_i_* and *η_i_* are species-level effects and *α_j_* and *β_j_* are site-level effects on occurrence and detection, respectively [52], [53], [82]. We also modeled a correlation (*ρ*) between occurrence and detection based on the assumption that both are affected by species abundance, such that more abundant species would likely be both easier to detect and more prevalent across the landscape, and vice versa [52], [54]. We further hypothesized that, despite our attempt to achieve independence among sampled sites (through separation in space or time), the occurrence of a species at a site might be affected by the occurrence of that species at neighboring sites, independently of modeled covariates (i.e., due to unmeasured environmental features or intrinsic processes such as animal movement behavior; [56], [59]). Preliminary analysis of our camera trap detections also indicated the potential for some spatial autocorrelation in site occurrences (Appendix S1). Such spatial autocorrelation could potentially bias inference, yet common tests of autocorrelation (e.g., spatial correlograms of model residuals) are difficult to apply given that our response variable of interest – species occurrence at a site – was only partially observed. We therefore extended our model to accommodate the possibility of spatial autocorrelation among sampling sites using an adaptation of the auto-logistic model described by Royle and Dorazio ([54]: 314–321; cf. [56], [83]). We defined a spatial neighborhood around each sampling site as a 5-km radius circle (i.e., an area of approximately 79 km^2^, assumed to encompass short-term movements of individual animals) and specified an auto-covariate, autocov*_j_*, such that the occurrence of species *i* at site *j* could be influenced by species *i*'s occurrence at all *g* sites within the neighborhood, with the magnitude of influence inversely proportional to the distance between the focal station and particular neighboring station (further details in Appendix S1).

The most general model of occurrence for species *i* at site *j* was therefore specified as:
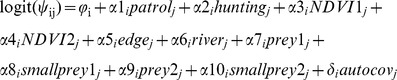
where *ϕ_i_* is a species-level effect, the coefficients *α*1*_i_*, *α*2*_i_*,…, *α*10*_i_* represent effects of the associated covariates (Table 1) on species *i*, and *δ_i_* is the effect of the autocovariate on species *i*. Similarly, the full detection model was specified as:



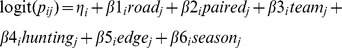
where *ηi* represents the species-level effect on detection and *β*1*_i_*,…, *β*6*_i_* are effects of the respective covariates on detection (details above).

Occurrence and detection processes were linked across species through the additional hierarchical model component in which species-level parameters were treated as random effects governed by community-level hyper-parameters. Specifically, we assumed that for a given effect (e.g., influence of patrol effort on occurrence), species-level parameters were drawn from a normal distribution described by the community mean (*μ*) and standard deviation (*σ*) hyper-parameters (e.g., *α*1*_i_* ∼ *N*(*μ_α_*_1_, *σ_α_*_1_)). We only considered additive, linear effects of covariates on occurrence and detection since we did not have strong *a priori* reasons for expecting non-linear or interactive effects and felt the additional model complexity was unwarranted given the available sample of observation data.

### Model selection

We considered all possible combinations of covariates to be candidate models representing competing hypotheses about significant influences on the MNP carnivore community (or its assessment in the case of detectability). Our *a priori* full model included 10 site-level covariates and an autocovariate for occurrence probability and 6 covariates for detection probability (yielding a candidate set with a daunting 2^17^ possible models). Given that several covariates represented similar features (e.g., 4 different prey indices), we anticipated that this model was likely overparameterized and therefore implemented a Bayesian approach to model simplification [84]. Information-theoretic approaches are commonly used to distinguish among competing models; for instance, the Akaike Information Criterion (AIC; [85]) or analogous Bayesian Deviance Information Criterion (DIC; [86]) balance model fit and complexity by ranking models using deviance and a penalty term weighted by the number of parameters. However, these criteria are not easily or reliably calculated for complex hierarchical models with latent variables, such as our multi-species occupancy model [87], [88]. For this reason, we used an alternative approach to model evaluation. We assessed the strength of evidence for covariate effects at the community-level (i.e., across all species) by estimating posterior model probabilities for the candidate set with a mixture modeling approach in which each covariate is multiplied by an “inclusion parameter” ([54]: 72–73, [84]: section 3.2, [89], [90]). The inclusion parameters (*w_c_*, for all *C* covariates in the model) were latent binary (Bernoulli) variables with uninformative prior probabilities of 0.5 (i.e., equal probability of a given covariate being included or not in the model). Their posterior probabilities corresponded to the estimated probability that a particular covariate was included in the “best” model; that is, the degree of support for an effect of that covariate across all carnivore species in the community. The posterior probability of a given candidate model (i.e., combination of covariate effects) was thus calculated as the probability that *w_c_*_ = _1 for all coefficients included in that model and *w_c_* = 0 for all coefficients not included. In other words, each of the 2^17^ candidate models had a corresponding unique vector of inclusion parameter values, and posterior probabilities for each of these vectors were calculated from their relative frequency in the posterior sample. For occurrence and detection parameters (i.e., *ϕ_i_*, *ηi*), posterior probabilities from the mixture model represented model-averaged estimates (i.e., averaged across the different models included in the posterior sample). Model-averaged estimates could also be obtained for covariate coefficients by averaging across posterior samples where the corresponding *w_c_*_ = _1 ([54]: 72–73).

Anticipating that different species may not show consistent responses, we also assessed the importance of covariates on individual species occurrence and detection probabilities by inspecting posterior distributions for all parameters from the full model (i.e., with no inclusion parameters, since these were only applied at the community-level). Species-level parameters (i.e., coefficients *α*1*_i_*, *α*2*_i_*, etc.) with posterior masses concentrated away from zero were considered indicative of an effect of the corresponding covariate on that particular species (e.g., zero not contained within credible intervals at 95%, or less conservatively, 80% probability thresholds).

We implemented all models in program WinBUGS version 1.4.3 [91], using the package R2WinBUGS [92] to interface with program R. Inference was made from 3,000 samples of the posterior distribution obtained from 3 chains of 50,000 Markov Chain Monte Carlo (MCMC) iterations after a burn-in of 50,000 and with a thin rate of 50. We used vague priors and random initial values, although achieving acceptable convergence in the MCMC chains required less diffuse prior specifications and other minor adjustments (sample code in Appendix S2; see also [54], [82]). Convergence was assessed by visual assessment of MCMC chains and using the Gelman-Rubin statistic (“Rhat” in R2WinBUGS, with values <1.1 indicating convergence; [76], [93]).

## Results

We detected nine carnivore species during the camera trap survey of 224 sites in MNP (Table 2). Spotted hyena (*Crocuta crocuta*) was detected at the greatest proportion of sampling sites (a “naïve” measure of occurrence without accounting for detectability; [50]), followed by leopard (*Panthera pardus*) and white-tailed mongoose (*Ichneumia albicauda*), whereas Gambian mongoose (*Mungos gambianus*) and side-striped jackal (*Canis adustus*) were detected at the fewest sites (Table 2). Model-estimated occurrence probabilities accounting for imperfect detection were higher than uncorrected estimates, but did not change the order of relative abundance across species. Species' occurrence and detection probabilities were significantly positively correlated (posterior mean of covariance parameter ρ = 0.47), suggesting both were related to underlying patterns of species abundance. The model-averaged community-level (i.e., across species) probabilities of site occurrence and per-survey detection were estimated to be 0.22 (posterior SD 0.09) and 0.12 (SD 0.04), respectively (based on the corresponding hyper-parameter posterior probabilities from the mixture model).

**Table 2:** Carnivore species detected during the camera trap survey in Mole National Park, Ghana, and estimated mean occurrence (*ψ*) and detection (*p*) probabilities and covariate effects on occurrence.

Common name^a^	Prop. sites	*ψ* (SD)	*p* (SD)	Covariate effects indicated^b^
Spotted hyena	0.442	0.544 (0.050)	0.173 (0.039)	small prey(+), riverine(+), edge(−), hunting(−), seasonal NDVI(+)
Leopard	0.299	0.526 (0.077)	0.140 (0.038)	small prey(+), riverine(+), patrol(−), hunting(+)
White-tailed mongoose	0.259	0.292 (0.039)	0.119 (0.031)	small prey(+), riverine(−), seasonal NDVI(−), patrol(−)
Large-spotted genet	0.246	0.263 (0.037)	0.146 (0.041)	small prey(+), edge(+), hunting(+), seasonal NDVI(+)
African civet	0.098	0.189 (0.062)	0.123 (0.047)	small prey(+)
Caracal	0.054	0.096 (0.045)	0.100 (0.047)	riverine(−), small prey(+)
Marsh mongoose	0.049	0.095 (0.053)	0.124 (0.060)	small prey(+)
Gambian mongoose	0.018	0.075 (0.073)	0.094 (0.053)	small prey(+)
Side-striped jackal	0.013	0.072 (0.089)	0.087 (0.054)	small prey(+)

The proportion of 224 sampling sites at which carnivore species were detected reflects observation data, whereas *ψ* and *p* are model-averaged estimates from the multi-species hierarchical mixture model (means and standard deviations from posterior probability distributions for species-specific parameters). Site covariates of occurrence are shown for cases where the posterior probability distribution from the full model for the corresponding species-specific coefficient indicated a potential effect (i.e., posterior mass not concentrated at 0; distributions are given in Appendix S3).

a Scientific names in Table S1.

b Direction of effect indicated as either positive (+) or negative (−) association of species occurrence probability with the particular covariate. For the different prey biomass covariates, only the strongest effect is indicated.

### Community-level covariate effects

Parameter estimates from our fully parameterized multi-species model were generally imprecise, with most posterior probabilities being widely distributed around their respective means and 95% CIs broadly overlapping 0 (Table 3, Appendix S3), implying that there was not a consistent response across the carnivore community to most site covariates. Posterior distributions for community-level hyper-parameters from the full model indicated the most consistent covariate effect on carnivore occurrence was a positive association with short-term or seasonal small prey biomass. There was also evidence of a consistent “observer effect” on detection probability (i.e., the “team” covariate), with higher mean community-level detectability associated with camera stations set by the primary sampling team (Table 3, Appendix S3).

**Table 3:** Posterior probability summaries of hyper-parameters for mean community-level effects of hypothesized site covariates on carnivore occurrence (*α* and *δ* coefficients) and detection (*β* coefficients).

Parameter (covariate)	Mean	SD	95% CI	Inclusion probability
*α*1 (patrol effort)	−0.19	0.29	−0.77, 0.39	0.219
*α*2 (hunting activity)	−0.04	0.32	−0.76, 0.56	0.015
*α*3 (seasonal NDVI)	0.04	0.25	−0.45, 0.51	0.028
*α*4 (integrated NDVI)	−0.08	0.20	−0.48, 0.32	0.001
*α*5 (edge distance)	−0.03	0.32	−0.67, 0.62	0.732
*α*6 (riverine distance)	−0.003	0.34	−0.69, 0.72	1.0
*α*7 (prey biomass, long-term)	0.13	0.29	−0.47, 0.65	*
*α*8 (small prey biomass, long-term)	0.33	0.26	−0.20, 0.81	*
*α*9 (prey biomass, short-term)	−0.26	0.31	−0.92, 0.33	0.010
*α*10 (small prey biomass, short-term)	1.18	0.40	0.51, 2.10	1.0
*δ* (spatial autocovariate)	0.76	1.12	−1.36, 3.28	0
*β*1 (road)	−0.12	0.43	−1.01, 0.69	0.910
*β*2 (paired stations)	0.10	0.27	−0.43, 0.63	0.011
*β*3 (set team)	−0.93	0.50	−2.08, −0.03	0.976
*β*4 (hunting activity)	−0.01	0.14	−0.27, 0.26	0.001
*β*5 (edge distance)	−0.16	0.29	−0.79, 0.34	0.479
*β*6 (season)	0.22	0.32	−0.42, 0.83	0.038

Posterior mean, standard deviation (SD) and 95% credible interval (CI) were estimated from the full model, while the corresponding inclusion probability from model selection using a mixture model is also shown (representing the posterior probability of that covariate effect being included in the best model). Posterior distributions for these hyper-parameters as well as species-level parameters are given in Appendix S3.

* The two prey indices derived from patrol data were not included in the final mixture model as they were considered redundant to (but less informative than) the comparable short-term prey indices derived from camera trap data (based on results of the full model and a preliminary mixture model).

**Figure 2:**
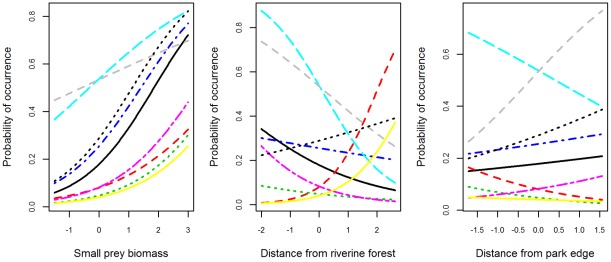
Model-predicted carnivore responses to the three site covariates included in the best occurrence model. Predicted marginal probabilities of carnivore occurrence relative to variation in the index of small prey biomass, distance from riverine forest, and distance from park edge (all values standardized). Species are: African civet (solid black), caracal (dashed red), Gambian mongoose (dotted green), large-spotted genet (dot-dash blue), leopard (dashed light blue), marsh mongoose (dot-dash purple), side-striped jackal (solid yellow), spotted hyena (dashed grey), white-tailed mongoose (dotted black; scientific names and details of model selection are given in the text).

Posterior probabilities for inclusion parameters on site covariates from the mixture model confirmed that small prey biomass was an important occurrence covariate for the MNP carnivore community (having an estimated probability of inclusion in the best model equal to 1; Table 3). They also highlighted the important community-level effect of proximity to riverine forest (mean posterior probability of inclusion, Pr = 1.0; Table 3), which was not apparent from the diffuse posterior of the full model due to the varied direction of species responses (Table 2; Fig. 2). There was some support for a community-wide edge effect on occurrence (Pr = 0.73, posterior SD = 0.44; Table 3), and weak evidence for a potential effect of patrol intensity (Pr = 0.22, SD = 0.41). Contrary to our primary hypothesis, there was little evidence of a consistent effect of hunting activity on carnivore occurrence, nor was there any indication of significant community-level effects of vegetation biomass (as measured by NDVI), total prey biomass, or spatial autocorrelation (Pr <0.03; Table 3).

With regard to carnivore detectability, the importance of the “team” covariate was strongly supported at the community-level by the posterior inclusion probability from the mixture model (Pr = 0.98, SD = 0.15; Table 3). A significant influence of roads was also indicated (Pr = 0.91, SD = 0.29), and there was limited support for an edge effect on detection probability (Pr = 0.48, SD = 0.50). Accordingly, combinations of these occurrence and detection covariates comprised the candidate models with the highest posterior model probabilities (Table 4). A total of 64 candidate models appeared in the posterior sample, but the four highest-ranked models had 70% of the support, and 90% of the posterior model probability was captured by 11 candidate models (Table 4). The top-ranked model contained additive effects of edge, riverine forest and small prey biomass on occurrence, and of road and team on detection (Pr = 0.335; Table 4). Predicted occurrence probabilities from the best model indicated significant heterogeneity among species in the direction and magnitude of their responses to site covariates (Fig. 2).

**Table 4:** Posterior model probabilities for the top 11 models that had 90% of the posterior support across all candidate models for community-level effects on carnivore occurrence (*ψ*) and detection (*p*), as estimated from the mixture modeling approach to model selection (53 additional models appeared in the posterior sample but all with probabilities <0.01).

Model	Posterior probability
*ψ*(edge + river + small prey) *p*(road + team)	0.335
*ψ*(river + small prey) *p*(road + team + edge)	0.139
*ψ*(edge + river + small prey) *p*(road + team + edge)	0.124
*ψ*(patrol + edge + river + small prey) *p*(road + team + edge)	0.103
*ψ*(patrol + edge + river + small prey) *p*(road + team)	0.043
*ψ*(river + small prey) *p*(road + team)	0.040
*ψ*(edge + river + small prey) *p*(team)	0.032
*ψ*(patrol + river + small prey) *p*(road + team + edge)	0.030
*ψ*(river + small prey) *p*(road + edge + season)	0.022
*ψ*(patrol + edge + river + small prey) *p*(team)	0.018
*ψ*(edge + river + small prey) *p*(team + edge)	0.016

### Species-level effects

We examined posterior probability distributions for all species-level parameters in the full model to identify potential species-specific effects that might be obscured at the community level. Posterior means for the effect of seasonal small prey biomass were positive for all 9 carnivore species, and 95% CIs overlapped 0 for only spotted hyena and caracal (*Caracal caracal*; Table 2, Appendix S3). There was weak species-level support for an effect of riverine forest habitat, both in terms of attraction (higher occurrence probabilities nearer to riverine forest for spotted hyena and leopard) and avoidance (lower occurrence probability near riverine forest for caracal; Fig. 2). The model indicated little evidence of an edge effect on occurrence probability for most species, although spotted hyena occurrence probability was marginally higher further away from the park edge, and the opposite was true for large-spotted genet (*Genetta pardina*; Fig. 2). Consistent with community-level estimates, there was little evidence for significant species-level effects of patrol effort, poaching activity, or vegetation biomass on carnivore occurrence, although some potential weak effects were indicated (Table 2, Appendix S3). In contrast, a signal of spatial autocorrelation in site occurrence probabilities was indicated for several species (i.e., positive posterior estimates of the autocovariate coefficient; Appendix S3).

In agreement with indications at the community-level, sampling-related heterogeneity in detection probabilities was evident at the species level. Posterior probabilities suggested most carnivore species had higher detectabilities at stations set by the primary sampling team (given occurrence), and that leopard and white-tailed mongoose were more likely to be detected at camera stations set on roads, whereas marsh mongoose was less likely to be detected on roads. Hunting activity and seasonality did not appear to affect species' detectabilities, but there was evidence of an edge effect, with posterior distributions for large-spotted genet, marsh mongoose (*Atilax paludinosus*) and spotted hyena suggesting lower detectability near the park edge, while those for leopard, Gambian mongoose and caracal indicated higher edge detectability.

## Discussion

### Factors influencing carnivore occurrence

Our results provide insight into the relative influence of anthropogenic and natural landscape features on the dynamics of a poorly studied carnivore community. The hierarchical multi-species modeling approach identified patterns across the entire community while also highlighting species-specific variation. Our models indicated that availability of suitable prey had the most consistent effect on the MNP carnivore community, with carnivore species' occurrence probabilities positively linked to the relative biomass of smaller prey species (particularly at a seasonal scale). While variation in vegetation biomass (as measured by NDVI) did not appear to significantly influence carnivore occurrence, our mixture model identified a key community-level effect of riverine corridors, reflecting an aggregate of varied species responses to this natural landscape feature. Contrary to expectation, heterogeneity in carnivore occurrence patterns was not associated with measured variation in illegal hunting activity, suggesting that hunting is not a dominant influence on carnivore species' use of park habitats (at least at the spatial and temporal scales examined). Our models did point to an effect of proximity to park edge on occurrence, implying that human disturbance may indeed exert influence on the carnivore community. However, this edge effect was not uniformly negative but rather highly variable across species (Fig. 2; unrelated to body mass or home range size), indicating that a simple model of increasing disturbance at the park edge is not appropriate.

Heterogeneity in species' responses to extrinsic stressors is to be expected, and consequently some inconsistency in aggregate responses interpreted at the community level should be anticipated (i.e., diffuse posterior distributions for community hyper-parameters). Nevertheless, uncertainty in our multi-species model also reflects the considerable amount of species-level variation in occurrence patterns unexplained by the spatial covariates we included (Appendix S3). Inference for rare species will always be limited by small sample sizes, and parameters were indeed less precisely estimated for carnivore species with few detections in our survey (e.g., Gambian mongoose, side-striped jackal; Appendix S3). All the same, the multi-species approach produced useful estimates of occurrence and detection probabilities for these species, and it is more powerful than single-species models that frequently fail to yield reliable estimates for rare species ([52], [94]; A.C. Burton unpublished data).

Even with the improved ability to estimate occurrence and detection probabilities, our modeling identified few effects of measured landscape covariates for the rarest carnivores in MNP (although some responses were strongly indicated, such as the negative association between caracal occurrence and proximity to riverine forest; Fig. 2). Responses to landscape factors were more discernible for species with a greater number of detections (e.g. spotted hyena, large-spotted genet; Appendix S3), and these likely had a significant influence on community-level inference. Since little is known about carnivore ecology in MNP, or more generally across much of West Africa [95], it is difficult to make a comparative assessment of the patterns of occurrence indicated by our study (particularly for smaller carnivores). Single-species studies from other areas agree with some of our findings while also highlighting the frequently complex relationships between landscape heterogeneity and carnivore ecology. For example, Marker and Dickman [96] found leopard abundance to be correlated with prey biomass (see also [25]), while Balme et al. [97] reported that leopards hunted preferentially in areas of intermediate vegetation cover where prey were easier to catch but not necessarily more abundant. Boydston et al. [98] and Kolowski and Holekamp [99] found that spotted hyenas selected areas with dense vegetation and near seasonal streams, but that their association with higher prey density was influenced by the degree of human disturbance. Negative edge effects on survival and behavior were reported for spotted hyenas [100] and leopards [11], although in the latter case leopards did not avoid edge areas (consistent with our results and perhaps indicative of an “ecological trap”, sensu [101]). There is a need to follow up on the results of our study with more detailed investigations of carnivore ecology in MNP (e.g., telemetry-based studies of habitat selection, survival and reproduction).

### Factors influencing carnivore detectability

Our hierarchical model also provided insight into biases associated with the sampling process. Firstly, detection probabilities per survey (i.e., per camera trap day) were estimated to be quite low, and accordingly our “naïve” estimates of occurrence probability were negatively biased by an average magnitude of 126% across all 9 carnivore species (from 7% for large-spotted genet to 434% for side-striped jackal, relative to model estimates; Table 2). This underscores the importance of accounting for imperfect detection in models of animal occurrence, a point which has been made previously by many authors (e.g., [50]) and yet has received relatively little attention in the broader literature on species distribution modeling [102], [103]. Explicit consideration of detectability is particularly important for rare and elusive species, such as most carnivores, and the largest estimated bias in our sample was associated with those species having the fewest detections (Table 2).

Our model indicated that two aspects of our sampling design introduced significant spatial heterogeneity to the probability of detecting a carnivore species given its occurrence. The potential bias of sampling on roads has been noted elsewhere [104], [105]. Yet, given access difficulties, we chose to set many camera stations at or near park roads (although roads in MNP are dirt tracks with relatively little vehicle traffic), and the explicit estimation of detection heterogeneity allowed us to address this sampling effect within the model. Similarly, despite our use of a standardized protocol for setting camera traps, we detected an “observer effect”, where detection probabilities differed between camera stations established by two field teams. Without an analytical method explicitly accounting for detectability, and recording of the relevant sampling covariate, this effect may have been erroneously interpreted as a difference in occurrence probability. The apparent influence of proximity to the park edge on detectability could be related to behavioral responses of carnivores to variation in human disturbance (e.g., increased vigilance in closer proximity to human settlement), and, if unaccounted for, may have distorted inference of edge effect on occurrence. Finally, our modeling results suggest that we adequately achieved independence among camera stations by separating them in space and time, since inclusion of the spatial autocovariate term was not supported at the community level. Nevertheless, posterior probability distributions for the autocovariate coefficient were suggestive of spatial autocorrelation in occurrence probabilities for several species (Appendix S3), so its potential importance should not be ignored in future work. Sampling design of future carnivore surveys in MNP (and elsewhere) will benefit from careful consideration of the detection biases indicated by our analysis.

### Study limitations

Limitations of our study that might affect the strength of inference must be carefully considered. Due to logistical constraints, we were unable to access many portions of the park or to implement a random sampling design, so our camera stations (and resulting detections) may represent a biased sample yielding incomplete information on carnivore occurrence patterns in relation to park features. Nevertheless, we were able to sample across gradients in our hypothesized factors of influence, and we attempted to control for the effects of spatial and temporal sampling features, such as roads and season, on detectability. We infrequently detected several of the carnivore species in MNP, a common challenge in surveys of rare and elusive species, and despite advantages of the multi-species modeling approach, stronger inference is ultimately achieved only by greater sampling effort (including more targeted, species-specific sampling).

Our indices representing anthropogenic and natural landscape features of hypothesized importance were generated from the best available information, but their reliability may be diminished by associated uncertainty. For instance, our measures of illegal hunting activity and longer-term prey biomass are dependent on the reliability of data generated by the patrol monitoring system, which is subject to an unknown amount of error [64] (see also [106]). Hunting pressure is particularly difficult to estimate given that hunters seek to avoid detection by patrols, thus accounting for hunter detectability is an important area for further research. Patrol data also underestimated the occurrence and abundance of smaller prey species ([64]; Table S1), so corresponding biomass indices are dominated by the larger and better-detected species. Prey indices derived from our camera-trap survey are subject to the same sampling limitations noted above for the carnivore data, and while the camera data included more small prey items (Table S1), the diet range for several of the smaller carnivores is poorly represented. Future work focusing more specifically on these smaller carnivores and their prey is therefore recommended.

Important variation in carnivore habitat quality may not have been adequately described by NDVI, which might be more tightly linked to the ecological characteristics of certain herbivores [70], [71]. While such remote sensing products show great promise for improving ecological understanding across large spatial and temporal scales [69], [107], they are not a substitute for detailed, field-based assessments of habitat that are largely lacking for MNP. Even an index as seemingly simple as distance to the park edge is subject to some uncertainty associated with inconsistent boundary demarcation [63], and its reliability as a proxy for human disturbance is affected by spatial variation in population density and land use around the park (A.C. Burton, unpublished data). Nevertheless, such limitations are common to many protected areas, particularly in developing nations like Ghana, and our study highlights a conservation-relevant approach to characterizing a park landscape. Future work should seek to test and improve upon these measures of landscape heterogeneity and address other important ecological factors (e.g., fire, [108]; competition, [109]) and modeling forms (e.g., interactive and non-linear effects; multi-scale occupancy for mobile species, cf. [110]).

### Conservation implications

MNP's historical carnivore community has been heavily impacted over recent decades, with the decline and potential extirpation of several species [18]. Assessing and maintaining the viability of persisting carnivore populations should therefore be of significant management concern, and our study provides useful information to that end. While illegal hunting pressure within the park is significant, we found no evidence that it exerts a direct influence on current spatial patterns of carnivore occurrence. Assuming this result to be accurate (i.e., not due to mismeasurement of hunting pressure), it could relate to the elusive nature of carnivores or the lack of hunter preference for these species. While there is evidence that many carnivore species are killed for local consumptive uses [18], MNP enforcement teams rarely report evidence of carnivore remains confiscated from arrested hunters (C. Balangtaa, pers. obs.). It is possible that carnivore species persisting in the park have proven themselves more resilient to direct human impacts like hunting, having passed through the “extinction filter” that apparently claimed other species [18], [111].

Assessing the indirect impacts of human activity on carnivore populations is more difficult. For instance, the positive association between carnivore occurrence and prey biomass is expected from natural predator-prey dynamics, but could also be indirectly influenced by hunting impacts on prey populations. Nevertheless, the relative dominance of prey availability on carnivore occurrence suggested by our model may be an encouraging reflection of the prominence of natural influences on the park's carnivore populations, and it provides a tangible target for park managers (i.e., protection of prey populations). Similarly, the lack of a strong or consistent edge effect on carnivore occurrence suggests that elevated impacts around the park are not undermining its effectiveness in protecting carnivore habitat, at least for most populations that persist (although we note that hunting is not limited to the park edge; Fig. 1C). Indeed, MNP appears to effectively protect natural habitats such as the riverine forest corridors that our modeling indicated to be of importance to carnivore occurrence patterns.

However, in assessing the effectiveness of MNP's protection of carnivore populations, it is important to note the uncertainty reflected in our results, which ultimately represent a fairly coarse and preliminary assessment. Several species were rarely detected in our survey, limiting inference on their dynamics and suggesting that they could be perilously close to local extinction. Even among the more frequently detected species, the long-term viability of their populations has not yet been appraised. In fact, preliminary mark-recapture estimates of population density for leopard and spotted hyena – two of the most frequently detected species in our survey – suggest that they persist at low abundances relative to conspecific populations (A.C. Burton, unpublished data). A reliable assessment of carnivore population viability in MNP, and a better understanding of the nature of human impacts on these populations, will require continued and detailed monitoring of species-specific occurrences and demographic rates.

Though further work is needed, our approach provides a valuable framework for the assessment of wildlife communities subject to anthropogenic impact. Few studies capitalize on the powerful information available across entire communities, despite the fact that many surveys generate data for a range of species. In particular, a rapidly growing number of camera-trap surveys produce data on many species, both rare and common, which may not be fully utilized as attention is typically focused on one or a few target species [41], [49], [112]. We have shown how such camera-trap data are well-suited to a multi-species hierarchical modeling framework, resulting in robust estimation of occurrence and detection probabilities across focal communities. We demonstrated that a community-level approach can facilitate inference on individual species while providing more comprehensive insight at a scale relevant to ecosystem-level management. Furthermore, we showed how data that may be readily available for many protected areas, such as patrol-based monitoring observations and remotely sensed vegetation indices, can be used to test hypotheses about relative influences on protected wildlife populations. This approach may be particularly valuable for guiding management efforts in developing nation parks that lack established research programs but face pressing conservation needs.

## Supporting Information

Table S1 **Scientific names and mean body mass for all species included in the study, with relative abundance for prey species detected by patrol and camera-trap surveys in Mole National Park, Ghana** (**2006–2009**)**.**
(PDF)

Table S2 **Variance inflation factors and correlation coefficients for covariates used in models of carnivore occurrence and detection probability.**
(PDF)

Appendix S1 **Further detail on the assessment of spatial autocorrelation in carnivore occurrence patterns.**
(PDF)

Appendix S2 **Example segments of WinBUGS model code for the hierarchical multi-species carnivore occurrence model.**
(PDF)

Appendix S3 **Posterior distributions for community-level hyperparameters and species-level parameters from the full multi-species occurrence model.**
(PDF)
