# Supplementary material for: Hierarchical Multi-Species Modeling of Carnivore Responses to Hunting, Habitat and Prey in a West African Protected Area
Source: PLoS One. 2012 May 30;7(5):e38007. doi: 10.1371/journal.pone.0038007 (PMC3364199; doi:10.1371/journal.pone.0038007)
Supplement: Table S1 — Scientific names and mean body mass for all species included in the study, with relative abundance for prey species detected by patrol and camera-trap surveys in Mole National Park, Ghana (2006–2009). (PDF) [file pone.0038007.s001.pdf]

Table S1. Scientific names and mean body mass for all species included in the study, with relative abundance for prey species detected by patrol and camera-trap surveys in Mole National Park, Ghana (2006-2009).

Scientific name	Common name	Body mass (kg)	Camera detections	Patrol count
**Potential prey species**				
Syncerus caffer	African buffalo	592.7	99	1692
Hippotragus equinus	Roan antelope	264.2	73	1557
Kobus ellipsiprymnus	Waterbuck	204.4	144	6155
Alcelaphus buselaphus	Hartebeest	160.9	253	5542
Phacochoerus africanus	Warthog	82.5	278	4716
Kobus kob	Kob	80.0	553	19621
Orycteropus afer	Aardvark	56.2	86	0
Tragelaphus scriptus	Bushbuck	43.3	345	2071
Papio anubis	Olive baboon	17.7	467	9589
Ourebia ourebi	Oribi	17.2	3	279
Sylvicapra grimmia	Grey Duiker	15.6	45	366
Hystrix cristata	Crested Porcupine	13.4	135	0
Cephalophus rufilatus	Red-flanked Duiker	12.1	64	217
Erythrocebus patas	Patas Monkey	8.0	66	2372
Colobus vellerosus	Geoffroy's black and white colobus	7.7	0	70
Thryonomys swinderianus	Marsh cane rat (grasscutter)	3.8	23	0
Chlorocebus sabaeus	Green Monkey	3.7	171	3580
Lepus saxatilis	Scrub Hare	2.6	52	0
Cricetomys gambianus	Giant Rat	1.3	2	0
Xerus erythropus	Striped Ground Squirrel	0.6	6	0
Galago senegalensis	Senegal Galago	0.2	3	0
Bucorvus abyssinicus	Abyssinian Ground Hornbill	4.0	30	0
Neotis denhami	Denham's Bustard	4.8	1	0
Lissotis melanogaster	Black-bellied Bustard	1.2	2	0
Streptopelia spp/*Turtur abyssinicus*	Dove species	0.1	32	0
Francolinus bicalcaratus	Double-spurred Francolin	0.4	41	0
Numida meleagris	Helmeted Guineafowl	1.3	168	0
Ptilopachus petrosus	Stone Partridge	0.2	28	0

Scientific name	Common name	Body mass (kg)
**Carnivore species**		
Crocuta crocuta	Spotted hyena	63.4
Ichneumia albicauda	White-tailed mongoose	3.6
Panthera pardus	Leopard	52.4
Genetta pardina	Large-spotted genet	2.0
Civettictis civetta	African civet	12.1
Atilax paludinosus	Marsh mongoose	3.6
Caracal caracal	Caracal	12.0
Mungos gambianus	Gambian mongoose	1.6
Canis adustus	Side-striped jackal	10.4
