# Supplementary material for: Hierarchical Multi-Species Modeling of Carnivore Responses to Hunting, Habitat and Prey in a West African Protected Area
Source: PLoS One. 2012 May 30;7(5):e38007. doi: 10.1371/journal.pone.0038007 (PMC3364199; doi:10.1371/journal.pone.0038007)
Supplement: Table S2 — Variance inflation factors and correlation coefficients for covariates used in models of carnivore occurrence and detection probability. (PDF) [file pone.0038007.s002.pdf]

Table S2. Variance inflation factors (VIF) and Spearman's rank correlation coefficient for variables included as covariates in models of carnivore occurrence and detection probability (see Methods and Table 1 of main text for descriptions of variables).

	VIF	NDVI1	NDVI2	Edge	Road	Paired	Team	Patrol	Hunting	River	Prey1	SmallPrey1	Prey2	SmallPrey2
NDVI1	1.752													
NDVI2	1.275	0.284												
Edge	3.134	-0.173	0.151											
Road	1.467	0.241	0.136	0.145										
Paired	1.062	0.063	0.034	0.031	0.178									
Team	2.533	0.224	-0.107	-0.469	-0.103	-0.072								
Patrol	3.113	0.085	-0.121	-0.245	-0.278	-0.008	-0.009							
Hunting	1.327	-0.346	-0.049	0.173	-0.244	-0.108	-0.237	-0.023						
River	1.429	-0.022	-0.318	-0.299	0.204	0.058	0.068	-0.012	-0.088					
Prey1	3.291	-0.334	-0.110	0.361	-0.301	-0.001	-0.246	0.516	0.285	-0.211				
SmallPrey1	2.120	-0.081	0.107	0.389	-0.144	0.075	-0.269	0.367	0.259	-0.250	0.565			
Prey2	1.946	-0.388	-0.066	0.185	-0.373	-0.113	-0.056	0.274	0.256	-0.169	0.496	0.308		
SmallPrey2	1.372	0.072	0.128	0.240	0.039	0.041	-0.197	0.151	-0.025	-0.189	0.222	0.177	0.333	
Season	2.434	-0.375	0.084	0.376	0.016	0.043	-0.718	0.006	0.192	-0.004	0.282	0.230	0.155	0.17
