# Supplementary material for: Hierarchical Multi-Species Modeling of Carnivore Responses to Hunting, Habitat and Prey in a West African Protected Area
Source: PLoS One. 2012 May 30;7(5):e38007. doi: 10.1371/journal.pone.0038007 (PMC3364199; doi:10.1371/journal.pone.0038007)
Supplement: Appendix S1 — Further detail on the assessment of spatial autocorrelation in carnivore occurrence patterns. (PDF) [file pone.0038007.s003.pdf]

**Appendix S1.** Further detail on the assessment of spatial autocorrelation in carnivore occurrence patterns.

Part I: Potential for spatial autocorrelation

We aimed to achieve spatial independence in our sampling design by spacing camera stations at intervals of approximately 1 km. Nevertheless, for logistical reasons some sites were within 1km of each other (including a small number of sites that were re-sampled with different stations in different years). Furthermore, endogenous ecological processes such as ranging or dispersal behavior could be expected to span across distances greater than 1 km for most carnivore species in MNP. For instance, estimated home range sizes average about 40 $km^2$ across the nine species detected (Burton 2010), indicating that one individual of a particular species could use habitats across several adjacent sites, thereby reducing the likelihood that nearby sites were independent samples of species occurrence.

On the basis of two types of preliminary evidence, we felt that including spatial autocorrelation in our model was warranted. Firstly, as part of a concurrent mark-recapture analysis of population density, we calculated that the mean maximum distance moved by 15 spotted hyena individuals (identified by spotting patterns) was approximately 5 km, with similar results for individually identified leopards. This suggested that the range of individual movements for the period over which a site was sampled could have encompassed several adjacent stations (at least for such larger species). Secondly, preliminary examination of species detections (i.e., raw observations of "presence-absence" at camera sampling sites) using spline correlograms (Bjørnstad & Falck 2001) and Moran's I correlograms (Dormann et al. 2007) suggested some evidence of spatial autocorrelation. For example, the two plots below (Fig. S1) show spline correlograms (with outer lines giving 95% pointwise bootstrap confidence intervals) for detections of spotted hyena and large-spotted genet as a function of distance between camera stations (in meters. Correlograms implemented in the R package *ncf*). The x-intercept is interpreted as the distance at which site occurrences are no more similar than expected by chance for the sample area. There is thus an indication of positive spatial autocorrelation over short distances, although it may not be significant as 95% CIs overlap 0.

**Figure S1.** Spline correlograms for detections of spotted hyena and large spotted genet at camera stations (i.e., presence-absence or "P/A"; plot details in preceding text).

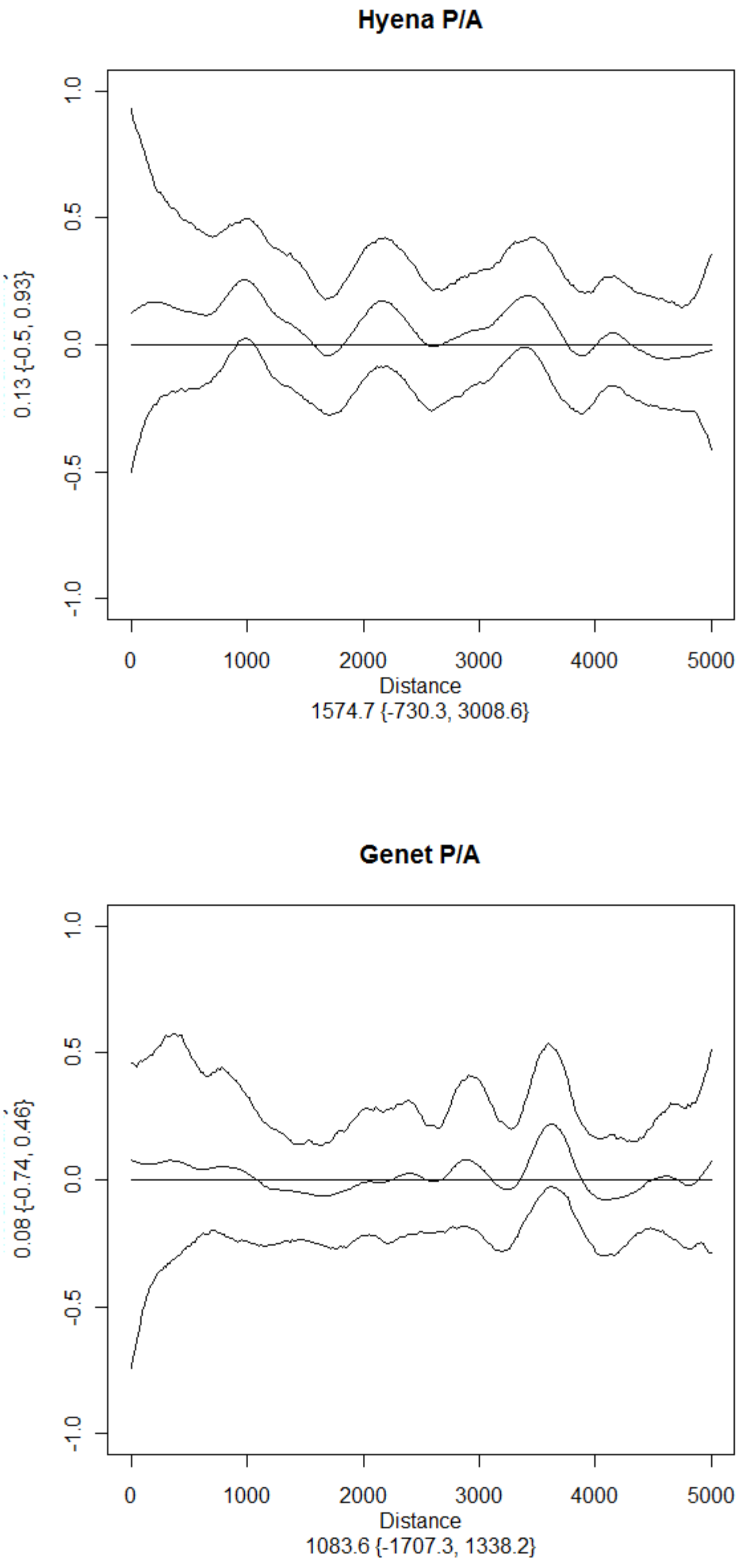

Part II: Neighborhood size

We initially defined four different sizes of spatial neighborhood as all stations within 1, 3, 5, or 10 km of a focal station, and compared the fit of multi-species models with each of the corresponding autocovariate terms as the only covariate (based on estimated deviance returned from R2WinBUGS). These distances represented neighborhood sizes of approximately 3, 28, 79 and 314 $km^2$, respectively, providing a gradient comparable to the range of home range sizes (and thus expected individual movements) for the nine carnivore species. Models with autocovariates based on a 5- and 10-km radii neighborhoods had similar estimated deviances which were substantially lower than those with 1- and 3-km radii neighborhoods. We used the 5-km radius (79 $km^2$) neighborhood for our autocovariate specification in subsequent modeling as we felt it represented a reasonable compromise for the varying home ranges sizes across the different species (Burton 2010). Further work could attempt a more systematic assessment of the effect of neighborhood size on the interpretation of spatial autocorrelation in this system.

Part III: Form of the modeled spatial autocovariate

Specification of the autocovariate for our hierarchical multi-species occurrence model is illustrated in the following segment of R code (for a 5-km radius spatial neighborhood):

```
  # load library spdep for neighborhood calculations and define X-Y coordinates
library(spdep)
coords <- as.matrix(cbind(site.cov$east/1000,site.cov$north/1000))
  # define neighbors at threshold distance = n.dist (in kilometers, UTM)
n.dist <- 5
nb.5km <- dnearneigh(coords,0,n.dist)
  # calculate vector numnn[j] specifying the number of neighbors of site j
numnn <- rep(0,J)
for (j in 1:J) numnn[j] <- length(nb.5km[[j]])
  # calculate distances for the neighbors
nb.5km.d <- nbdists(nb.5km, coords)

  # Construct matrix NN[j,g] which identifies the G neighbors of site j
NN <- matrix(rep(0,J*max(numnn)),nrow=J)
for (j in 1:J) {
      NN[j,] <- append(as.vector(nb.5km[[j]]),rep(NA,max(numnn)-numnn[j]))
      }

  # Construct matrix D[j,g] specifiying the distance between site j and …
      # … neighboring site g (with NAs filled in as needed)
D <- matrix(rep(0,J*max(numnn)),nrow=J)
for (j in 1:J) {
      D[j,] <- append(as.vector(nb.5km.d[[j]]),rep(NA,max(numnn)-numnn[j]))
      }
```

## Literature Cited

Bjørnstad O.N. & Falck W. 2001. Nonparametric spatial covariance functions: Estimation and testing. *Environmental and Ecological Statistics*, 8, 53-70.

Burton, A. C. 2010. Wildlife monitoring and conservation in a West African protected area. PhD dissertation. University of California, Berkeley. 149 p.

Dormann C.F., McPherson J.M., Araujo M.B., Bivand R., Bolliger J., Carl G., et al. 2007. Methods to account for spatial autocorrelation in the analysis of species distributional data: a review. *Ecography*, 30, 609-628.
