# Supplementary material for: Hierarchical Multi-Species Modeling of Carnivore Responses to Hunting, Habitat and Prey in a West African Protected Area
Source: PLoS One. 2012 May 30;7(5):e38007. doi: 10.1371/journal.pone.0038007 (PMC3364199; doi:10.1371/journal.pone.0038007)
Supplement: Appendix S2 — Example segments of WinBUGS model code for the hierarchical multi-species carnivore occurrence model. (PDF) [file pone.0038007.s004.pdf]

Appendix S2. Example segments of WinBUGS model code for the hierarchical multi-species carnivore occurrence model (a "#" precedes annotation remarks. Refer to the Methods section of the main text for symbol equations and parameter definitions).

```
# specify prior probabilities
   # include prior on covariate inclusion parameters for mixture model, w[c] ~ dbern(0.5)
psi.mean ~ dunif(0,1)
alpha <- log(psi.mean) - log(1-psi.mean)

mu.delta ~ dunif(-5,5)
sigma.delta ~ dunif(0,5)
tau.delta <- (1/(sigma.delta*sigma.delta))

mu.a1 ~ dunif(-5,5)
tau.a1 ~ dgamma(0.1,0.1)
# … same specification for all 10 occurrence covariate coefficients
mu.b1 ~ dunif(-5,5)
tau.b1 ~ dgamma(0.1,0.1)
# … same specification for all 6 detection covariate coefficients

p.mean ~ dunif(0,1)
beta <- log(p.mean) - log(1-p.mean)

sigma.u ~ dunif(0,10)
sigma.v ~ dunif(0,10)
tau.u <- pow(sigma.u,-2)
tau.v <- pow(sigma.v,-2)
rho ~ dunif(-1,1)
var.eta <- tau.v/(1.-pow(rho,2))

# species-level parameters for n species. Note truncation trick for MCMC convergence

for (i in 1:n) {
    phi[i] ~ dnorm(alpha, tau.u)I(-10,10)
    mu.eta[i] <- beta + (rho*sigma.v/sigma.u)*(phi[i] - alpha)
    eta[i] ~ dnorm(mu.eta[i], var.eta)I(-10,10)
    delta[i] ~ dnorm(mu.delta, tau.delta)I(-10,10)
    a1[i] ~ dnorm(mu.a1, tau.a1)I(-5,5)
# … same basic specification for all 10 occurrence covariate coefficients
    b1[i] ~ dnorm(mu.b1, tau.b1)I(-5,5)
# … same basic specification for all 6 detection covariate coefficients
```

```
# specify autocovariate and occurrence and detection functions across J sites
       # note that inclusion parameters w[c] would be included on regression coefficients …
       # … for mixture model selection approach, e.g., w[1]*a1[i]*patrol[j]

  for (j in 1:J) {

   x[i,j,1] <- 0
       for (g in 1:numnn[j]) {
              x[i,j,g+1] <- x[i,j,g] + Z[i,(NN[j,g])]/D[j,g]
       }

    lpsi[i,j] <- phi[i] + a1[i]*patrol[j] + a2[i]*hunting[j] +
       a3[i]*ndvi1[j] + a4[i]*ndvi2[j] + a5[i]*edge[j] + a6[i]*river[j] + a7[i]*prey1[j] +
       a8[i]*smallprey1[j] + a9[i]*prey2[j] + a10[i]*smallprey2[j] +
       delta[i]*(x[i,j,numnn[j]+1]/numnn[j])

       psi[i,j] <- 1/(1+exp(-lpsi[i,j]))

       Z[i,j] ~ dbern(psi[i,j])

    lp[i,j] <- eta[i] + b1[i]*road[j] + b2[i]*paired[j] + b3[i]*team[j] + b4[i]*hunting[j]
       + b5[i]*edge[j] + b6[i]*season[j]

       p[i,j] <- 1/(1+exp(-lp[i,j]))

       mu.p[i,j] <- p[i,j]*Z[i,j]

       Y[i,j] ~ dbin(mu.p[i,j], K[j])
    }
}
# calculate site-specific estimate of species richness
for (j in 1:J) {
       Nsite[j] <- sum(Z[1:n,j])
}}
```
