# Supplementary material for: Hierarchical Multi-Species Modeling of Carnivore Responses to Hunting, Habitat and Prey in a West African Protected Area
Source: PLoS One. 2012 May 30;7(5):e38007. doi: 10.1371/journal.pone.0038007 (PMC3364199; doi:10.1371/journal.pone.0038007)

Appendix S3. Posterior distributions from the full model for community-level hyperparameters on occurrence and detection probabilities, and species-level parameters on occurrence probability (scientific names of species in Table S1).

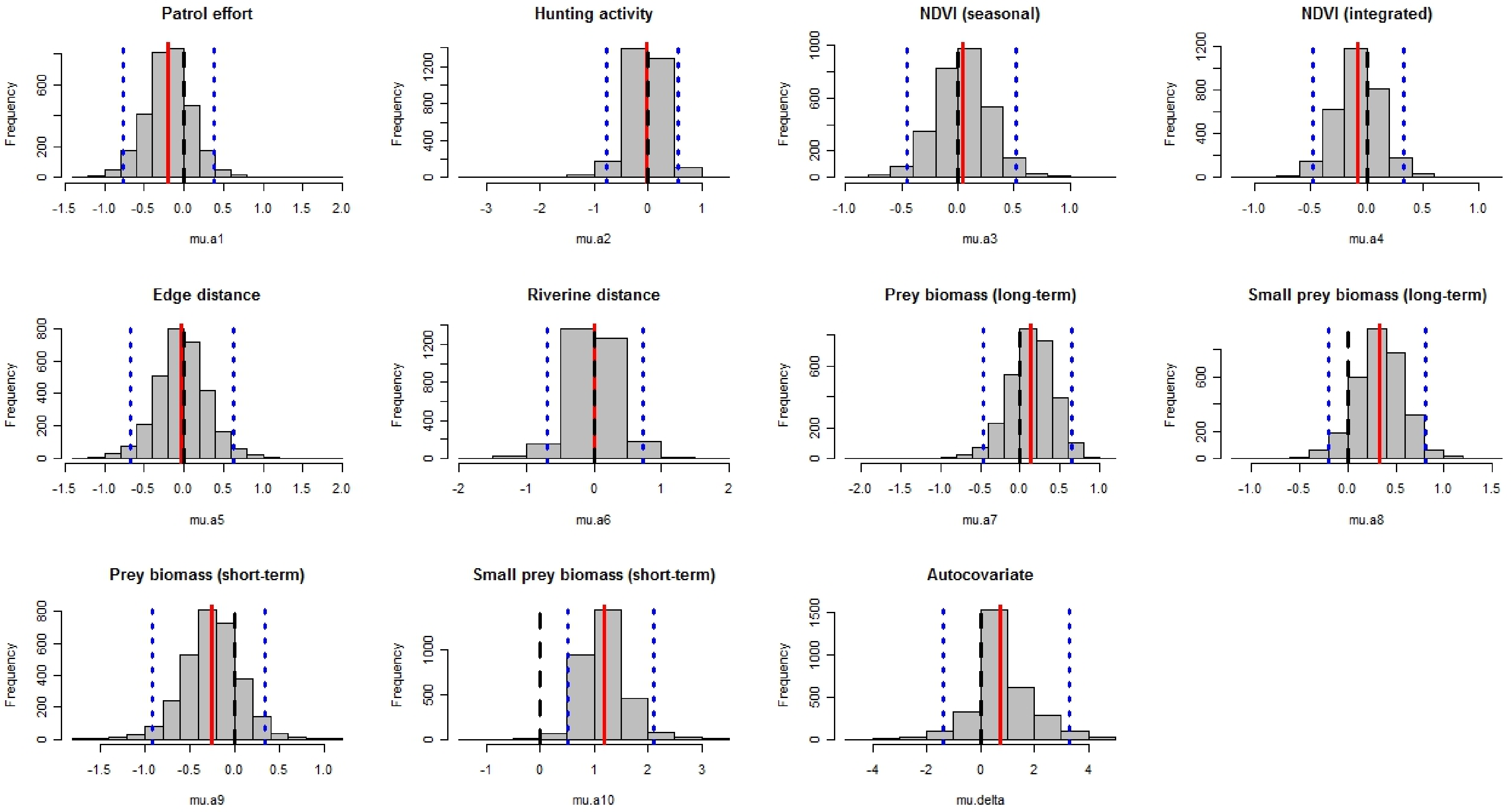

Posterior probability distributions for mean **community-level hyperparameters corresponding to site-level covariates on <u>occurrence</u>** probability. Vertical lines indicate 0 (i.e., no effect; black long-dash), the hyperparameter mean (red solid) and the 95% credible interval (blue short-dash).

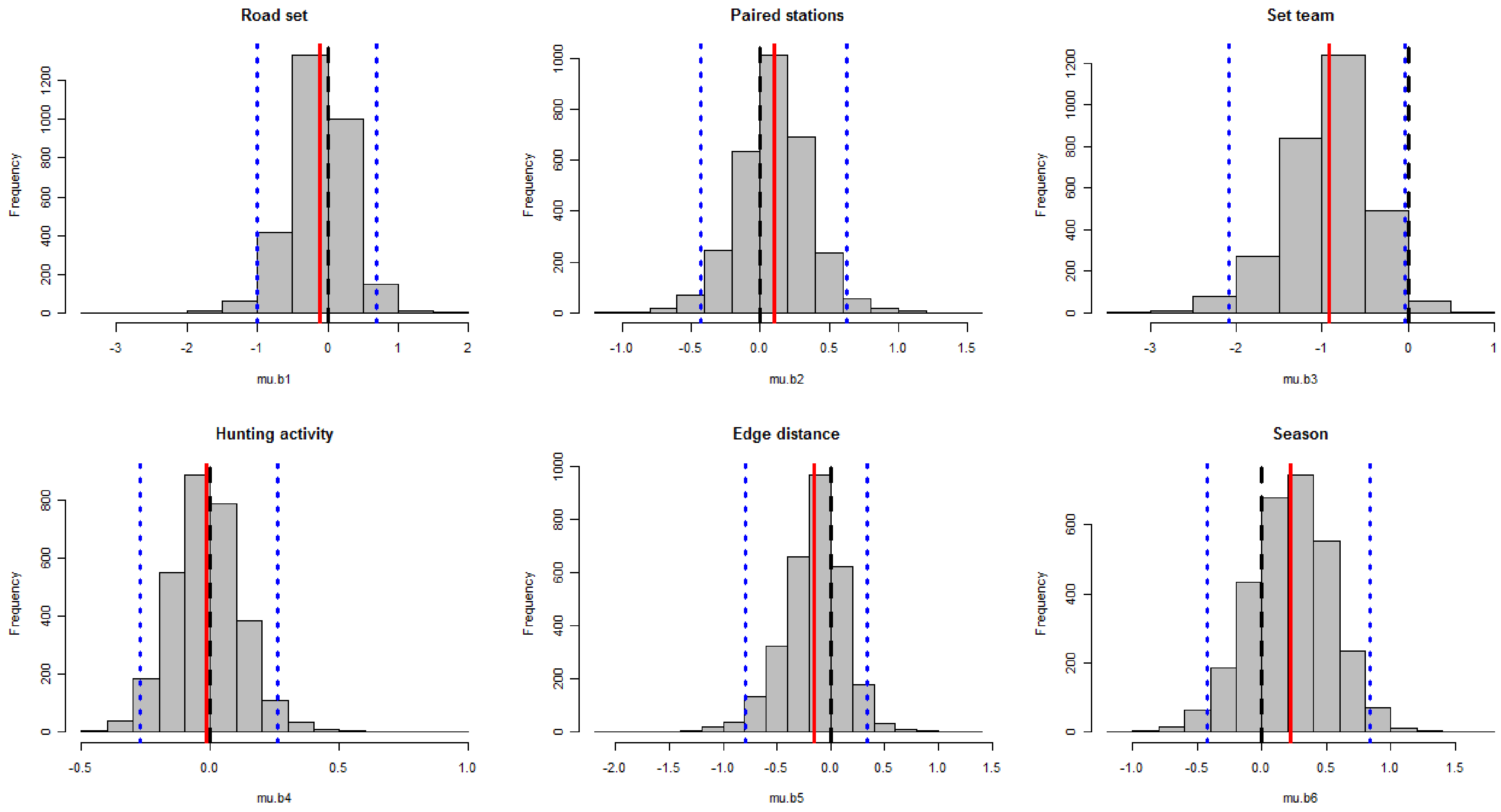

Posterior probability distributions for mean **community-level hyperparameters corresponding to site-level covariates on <u>detection</u>** probability. Vertical lines indicate 0 (i.e., no effect; black long-dash), the hyperparameter mean (red solid) and the 95% credible interval (blue short-dash).

Posterior probability distributions from full model for species-level occurrence coefficients on effect of **patrol effort** ($\alpha 1_i$; vertical line at 0, i.e., no effect)

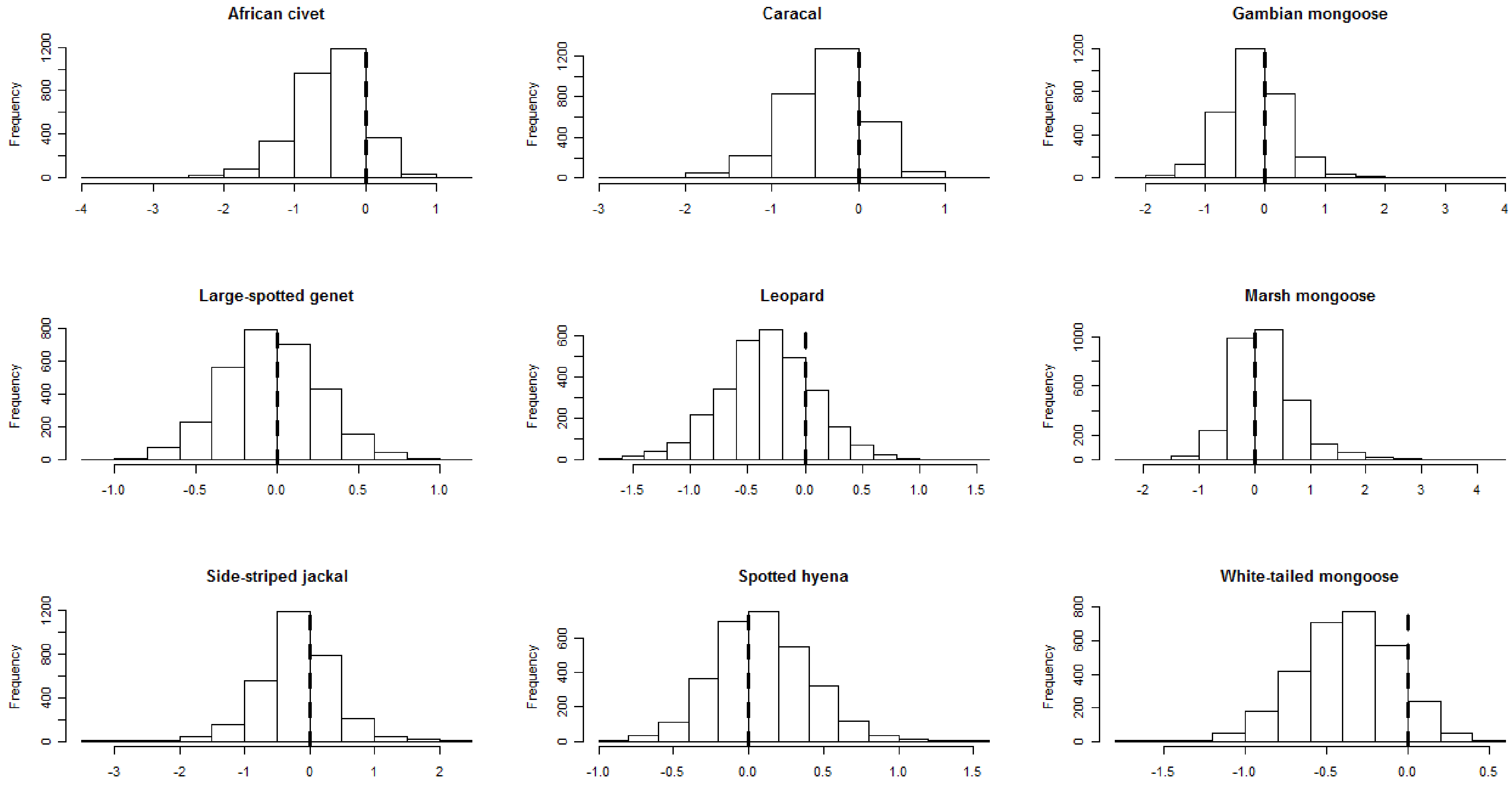

Posterior probability distributions from full model for species-level occurrence coefficients on effect of **hunting activity** ($\alpha 2_i$)

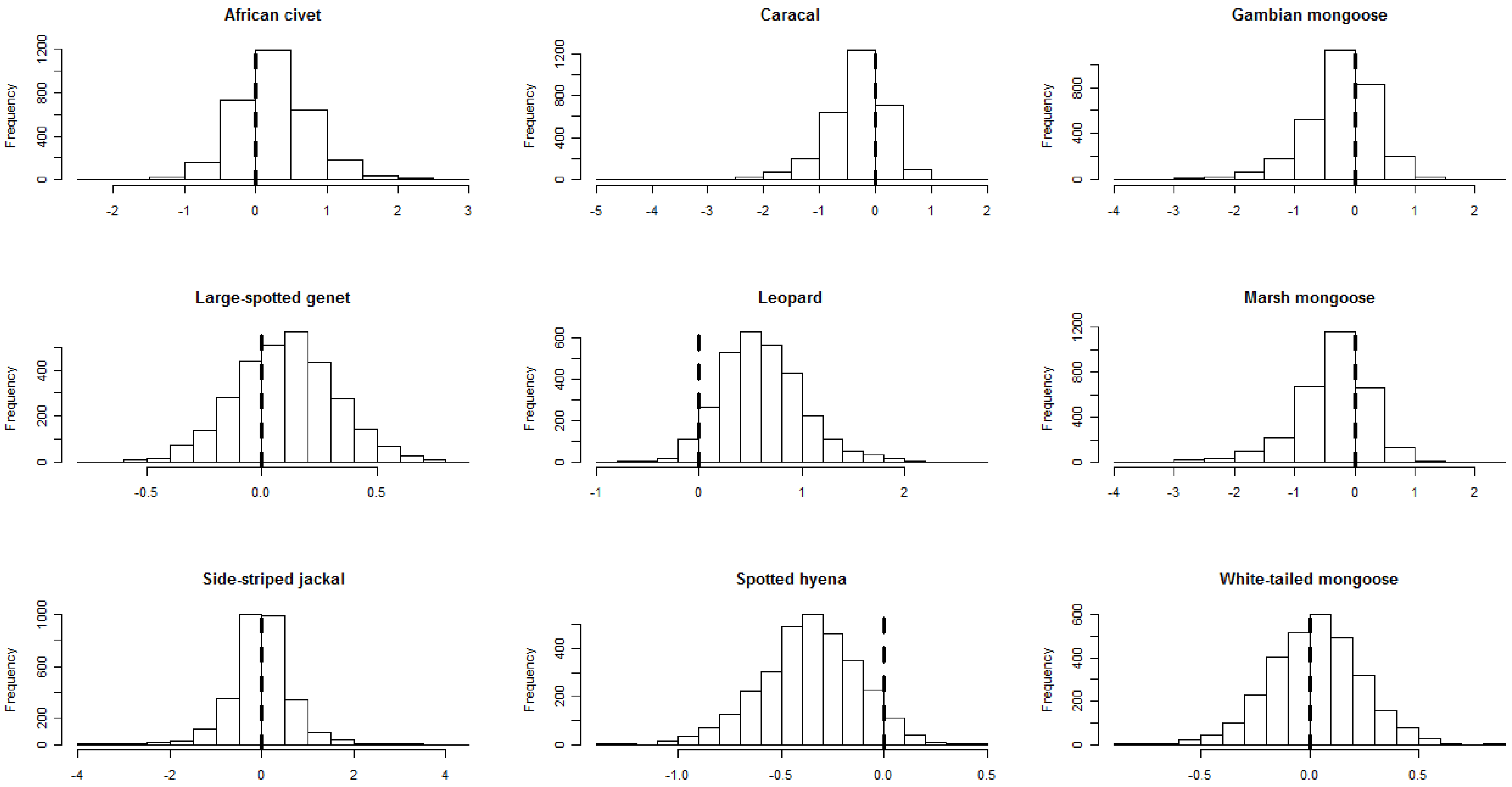

Posterior probability distributions from full model for species-level occurrence coefficients on effect of **distance from park edge** ($\alpha 5_i$)

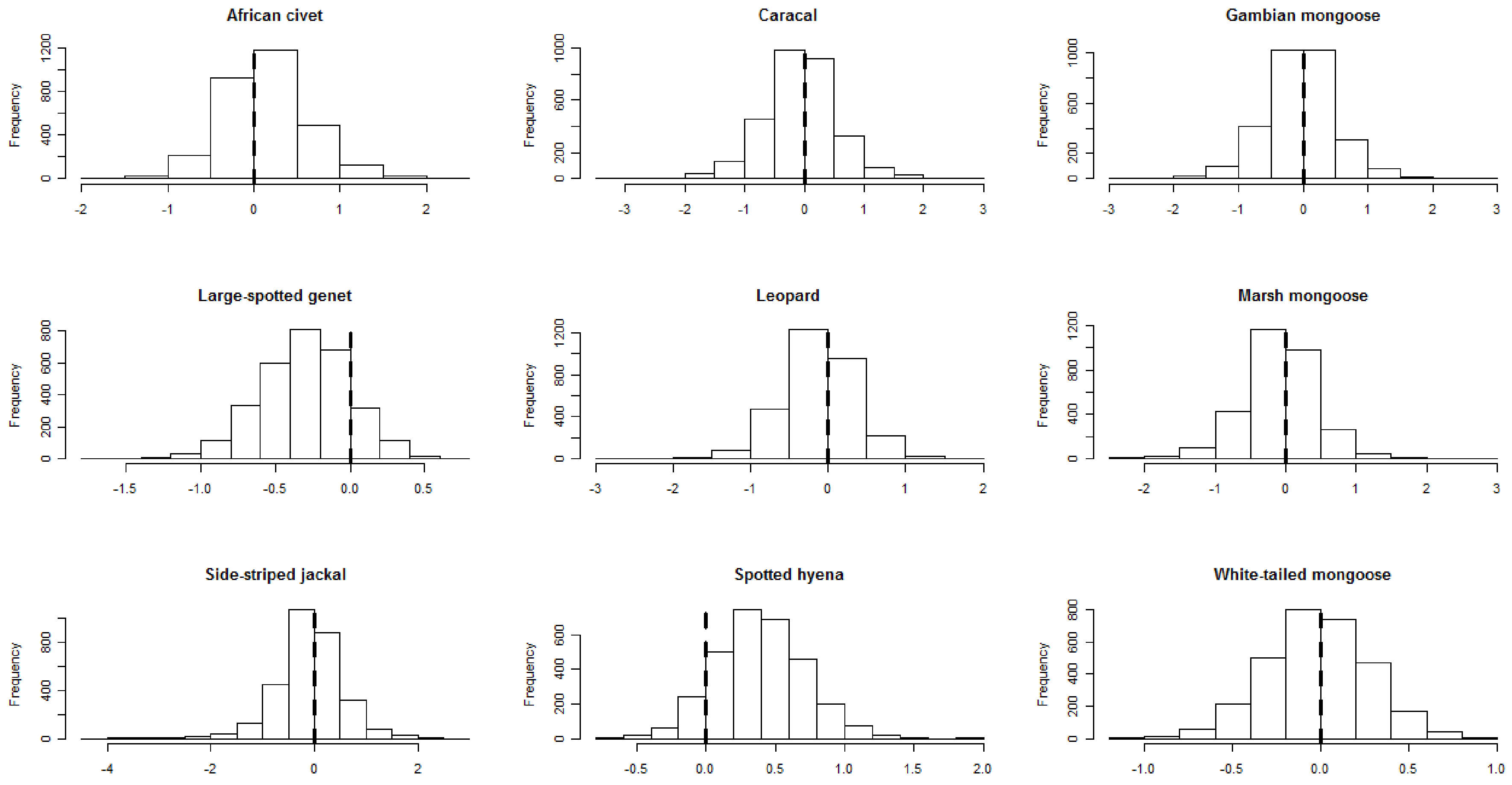

Posterior probability distributions from full model for species-level occurrence coefficients on effect of **distance from riverine forest** ($\alpha 6_i$)

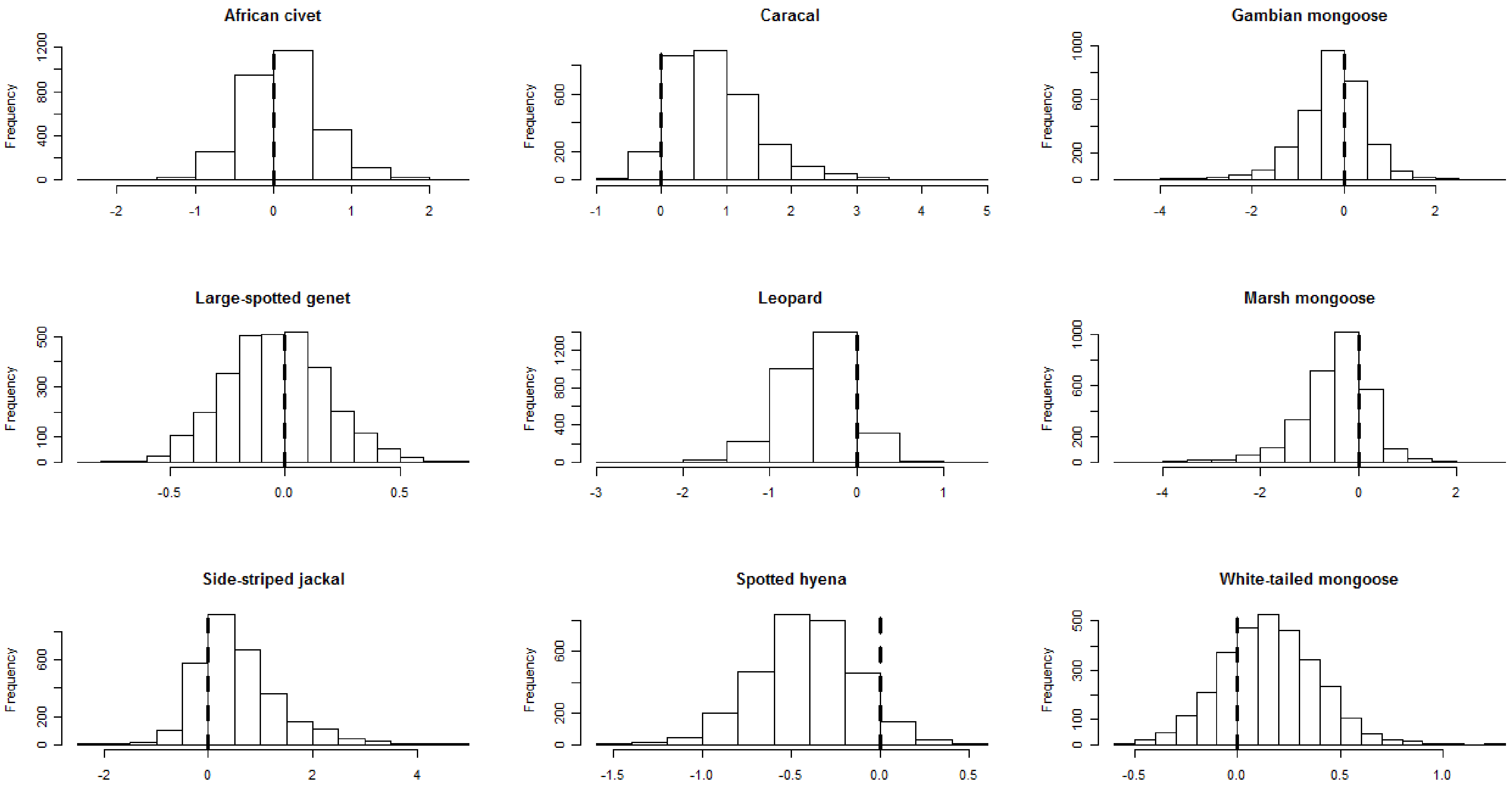

Posterior probability distributions from full model for species-level occurrence coefficients on effect of **<u>longer-term prey biomass</u>** ($\alpha 7_i$)

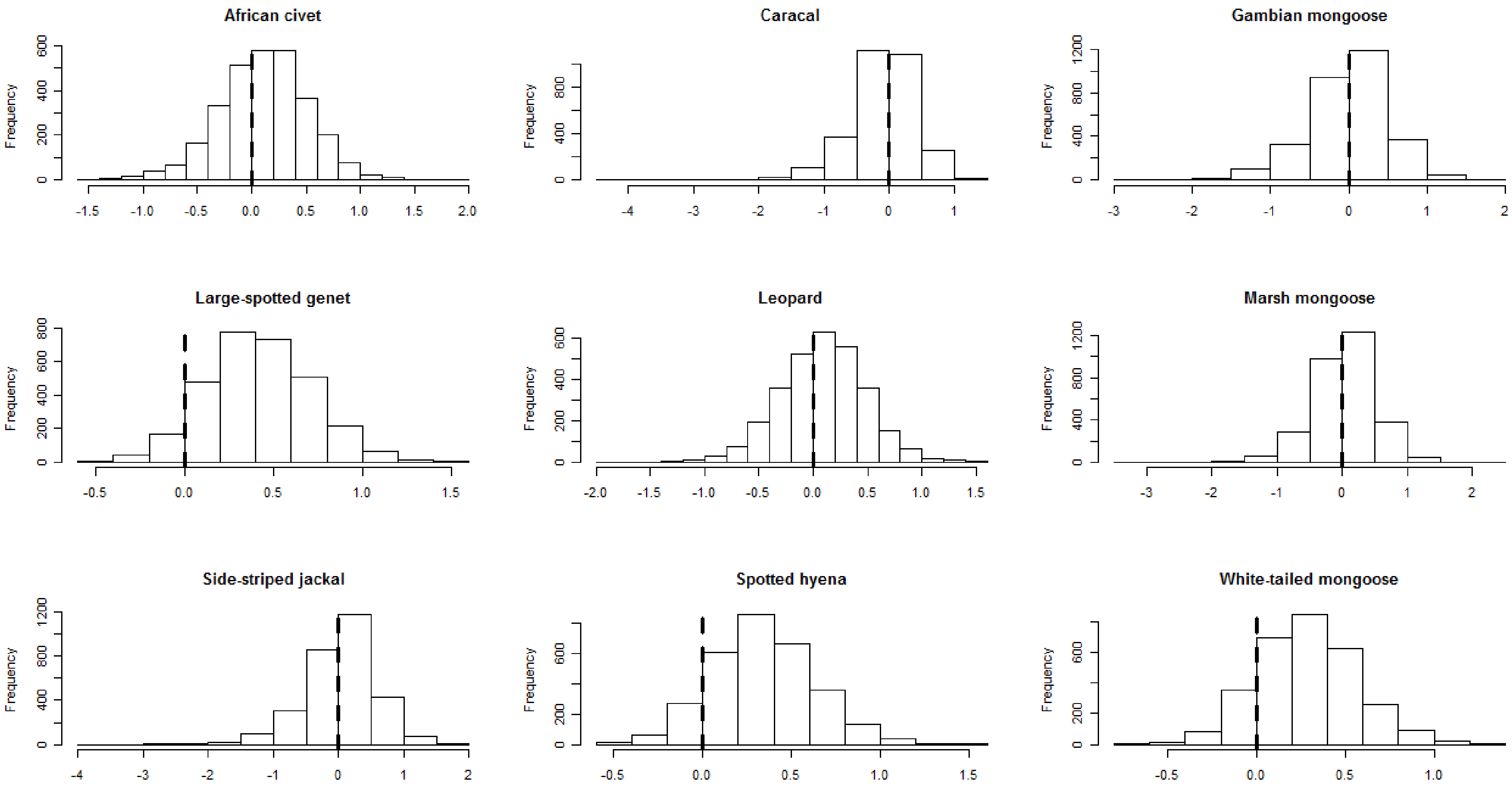

Posterior probability distributions from full model for species-level coefficients on effect of **<u>longer-term small prey biomass</u>** ($\alpha8_i$)

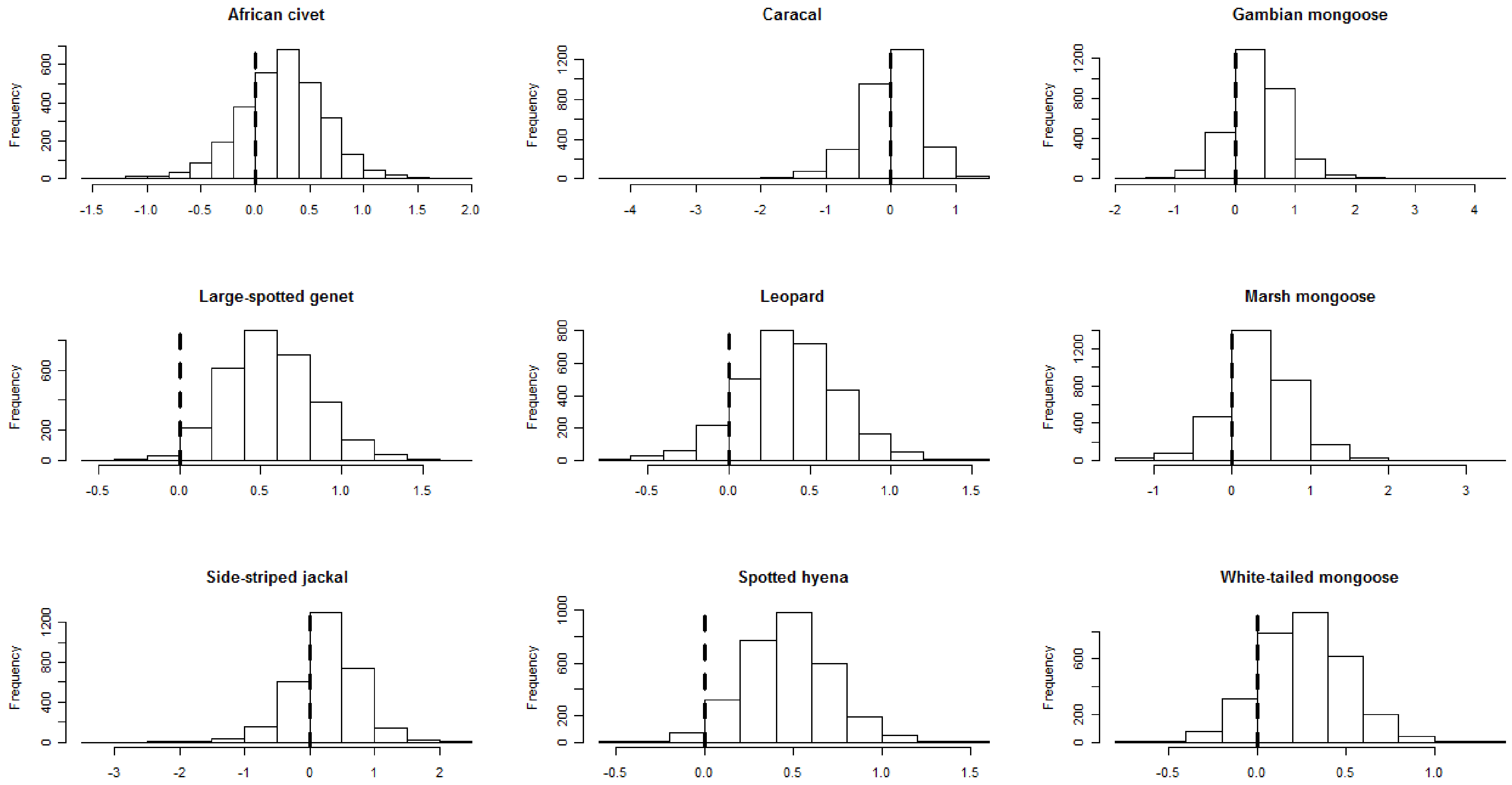

Posterior probability distributions from full model for species-level occurrence coefficients on effect of **seasonal prey biomass** ($\alpha 9_i$)

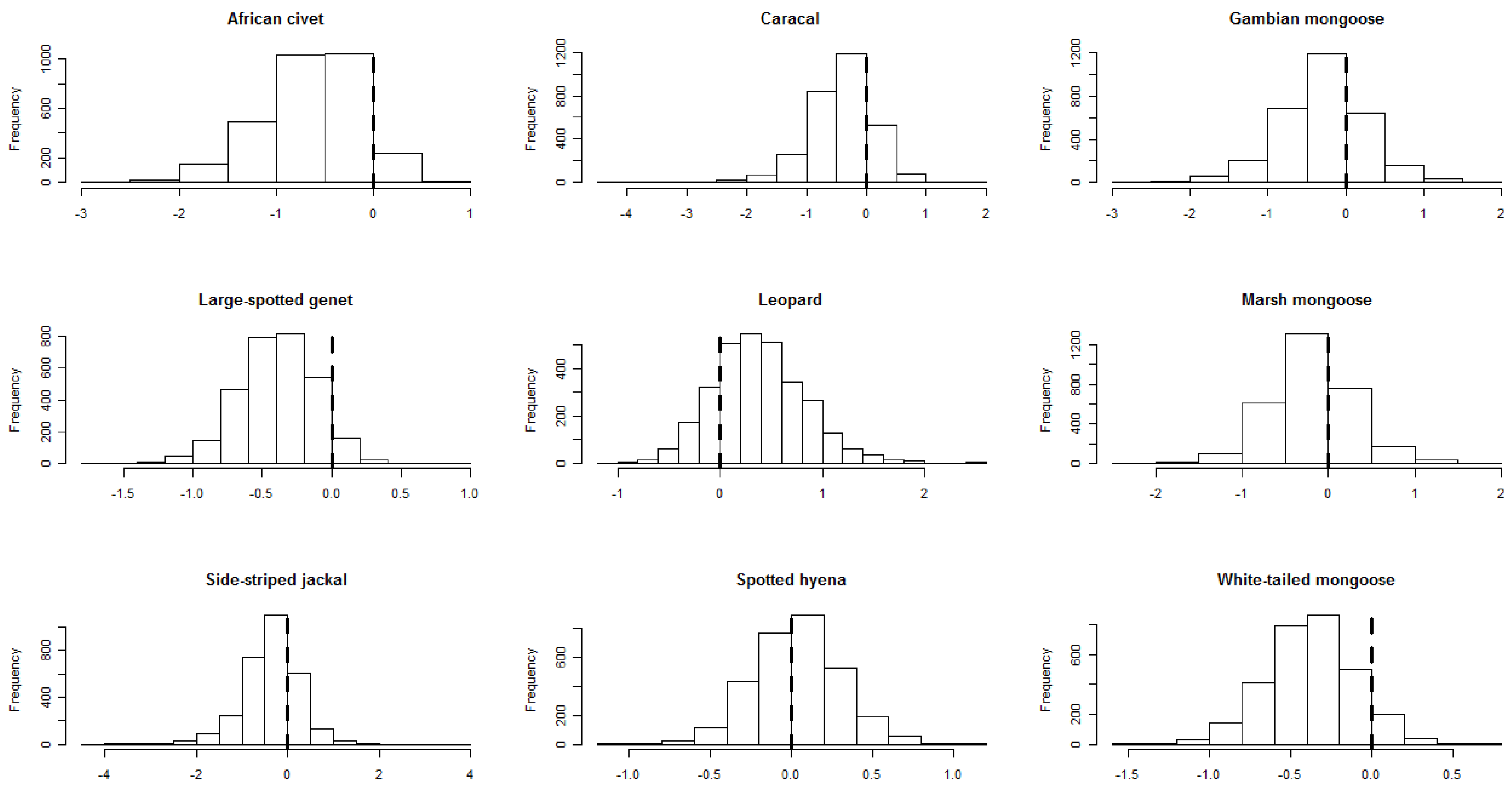

Posterior probability distributions from full model for species-level occurrence coefficients on effect of **seasonal small prey biomass** ($\alpha 10_i$)

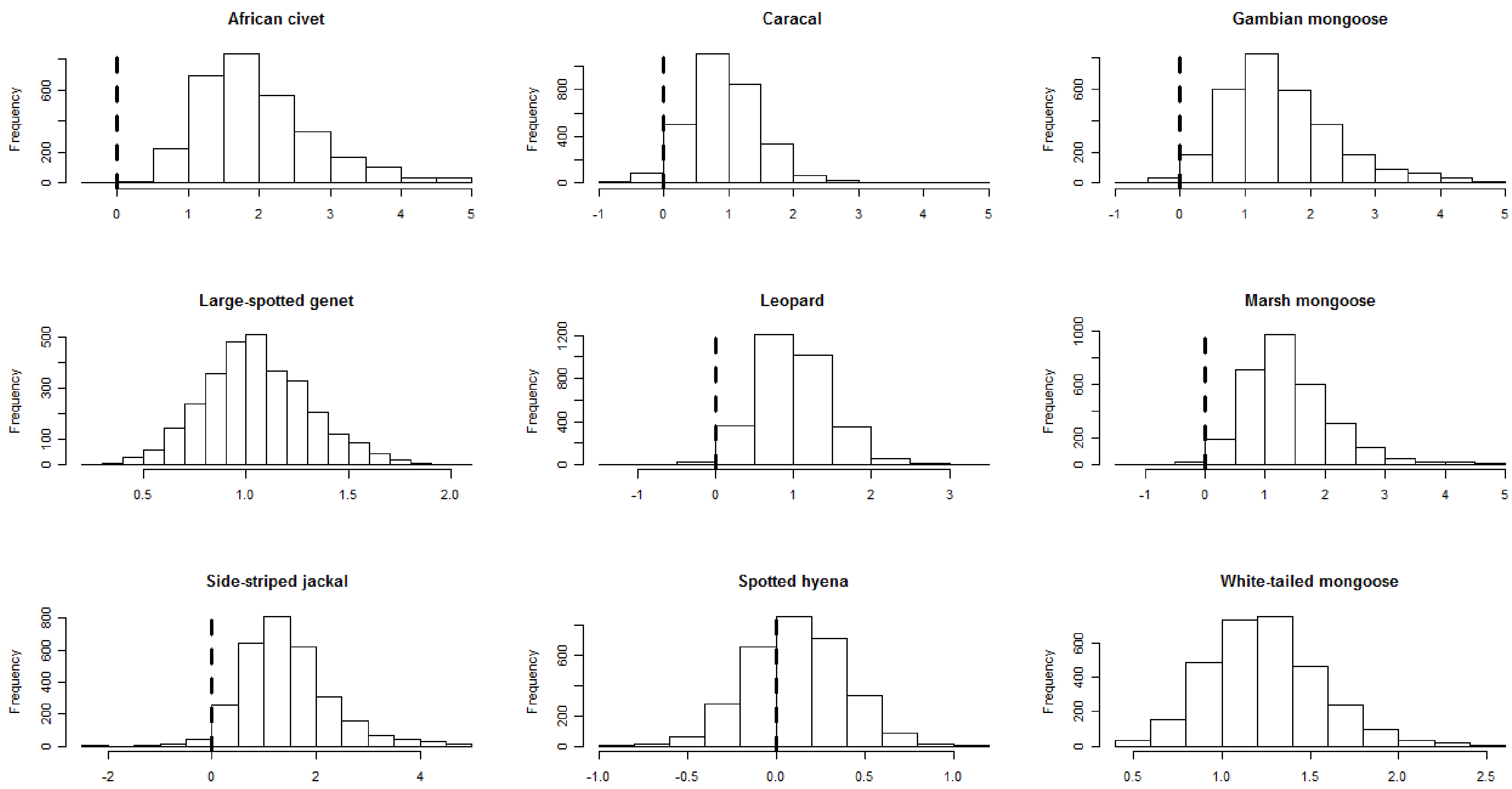

Posterior probability distributions from full model for species-level occurrence coefficients on effect of **seasonal NDVI** ($\alpha 3_i$)

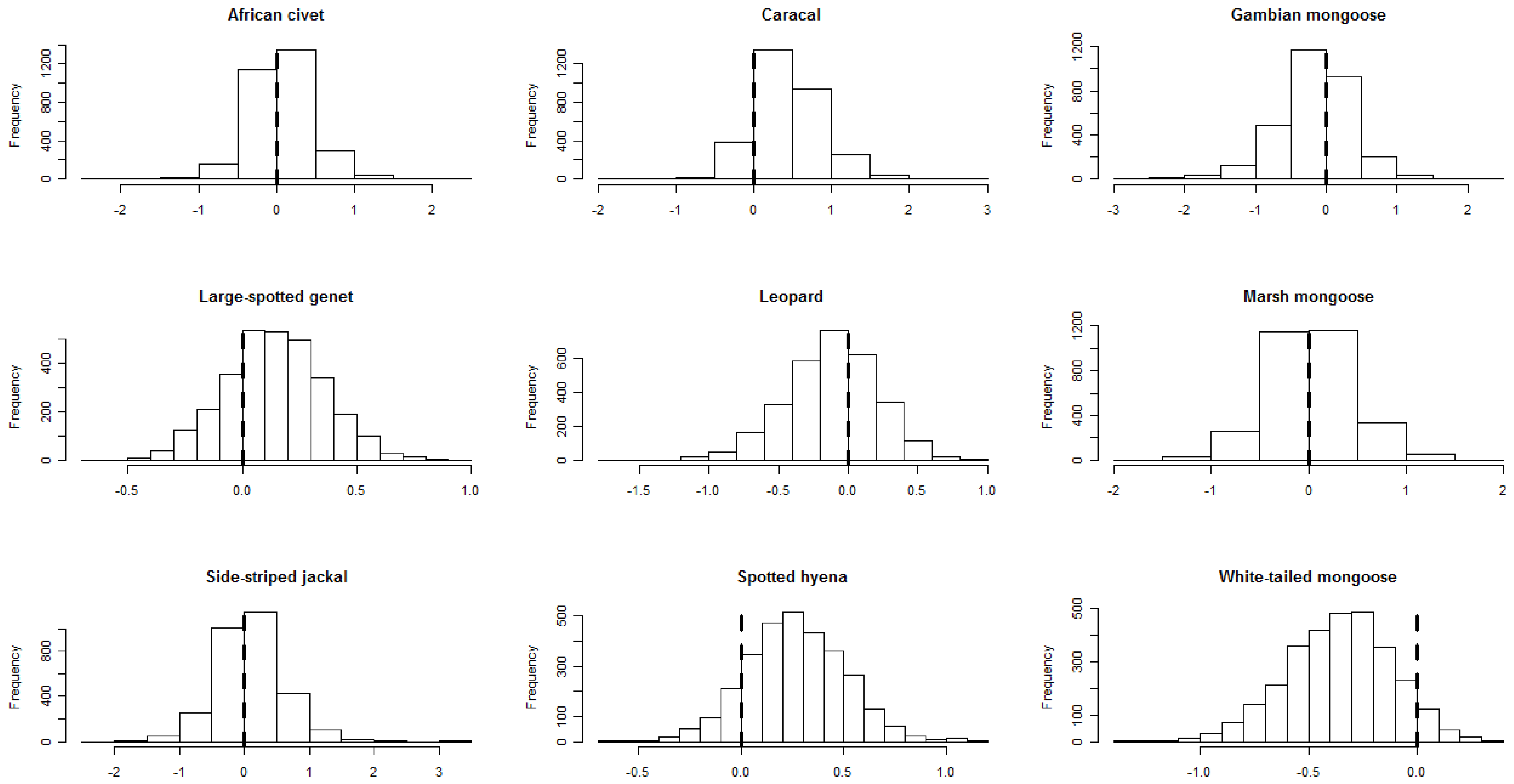

Posterior probability distributions from full model for species-level occurrence coefficients on effect of **integrated NDVI** ($\alpha 4_i$)

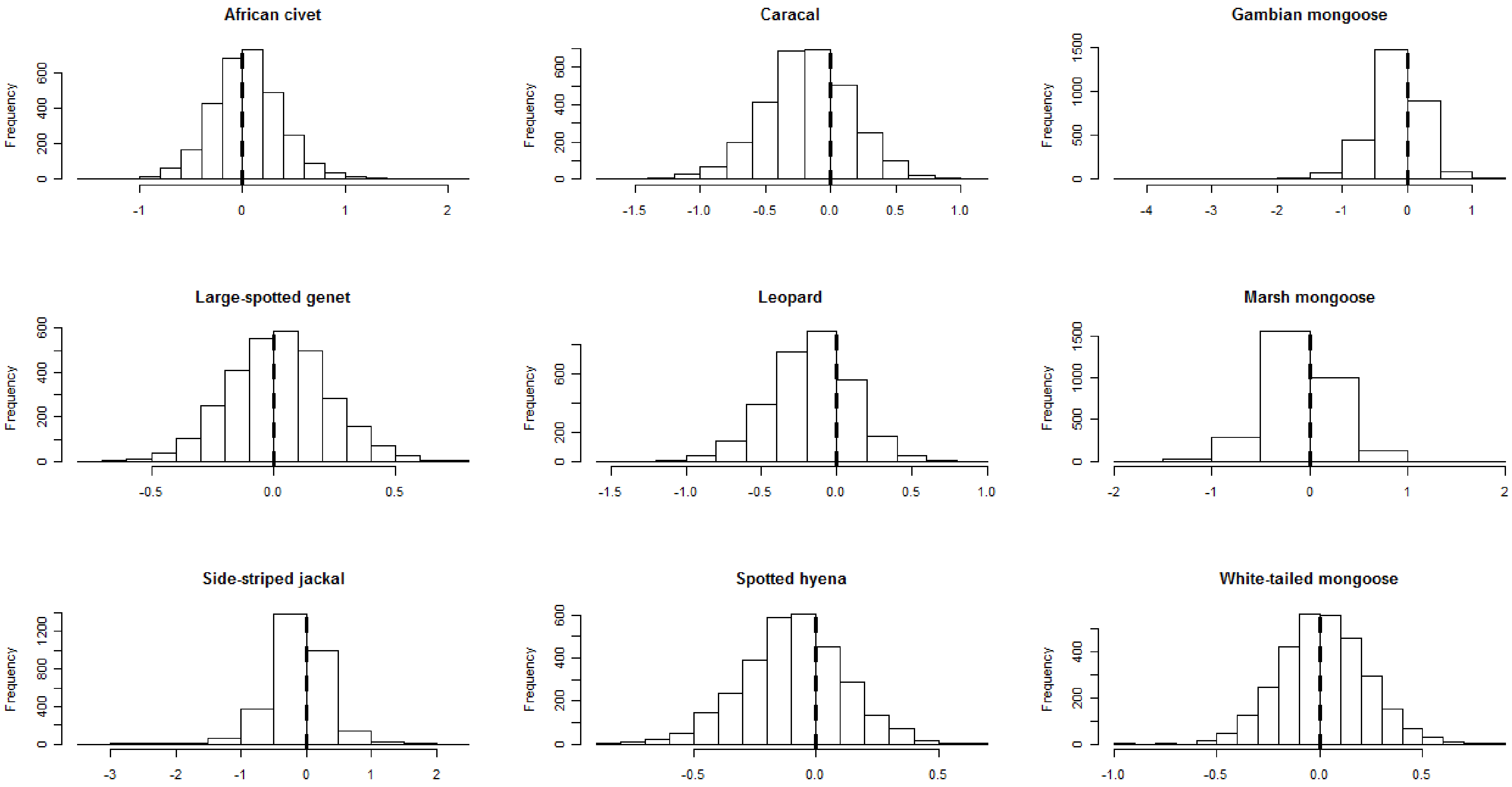

Posterior probability distributions from full model for species-level coefficients on effect of **<u>spatial autocovariate</u>** (δ_i)

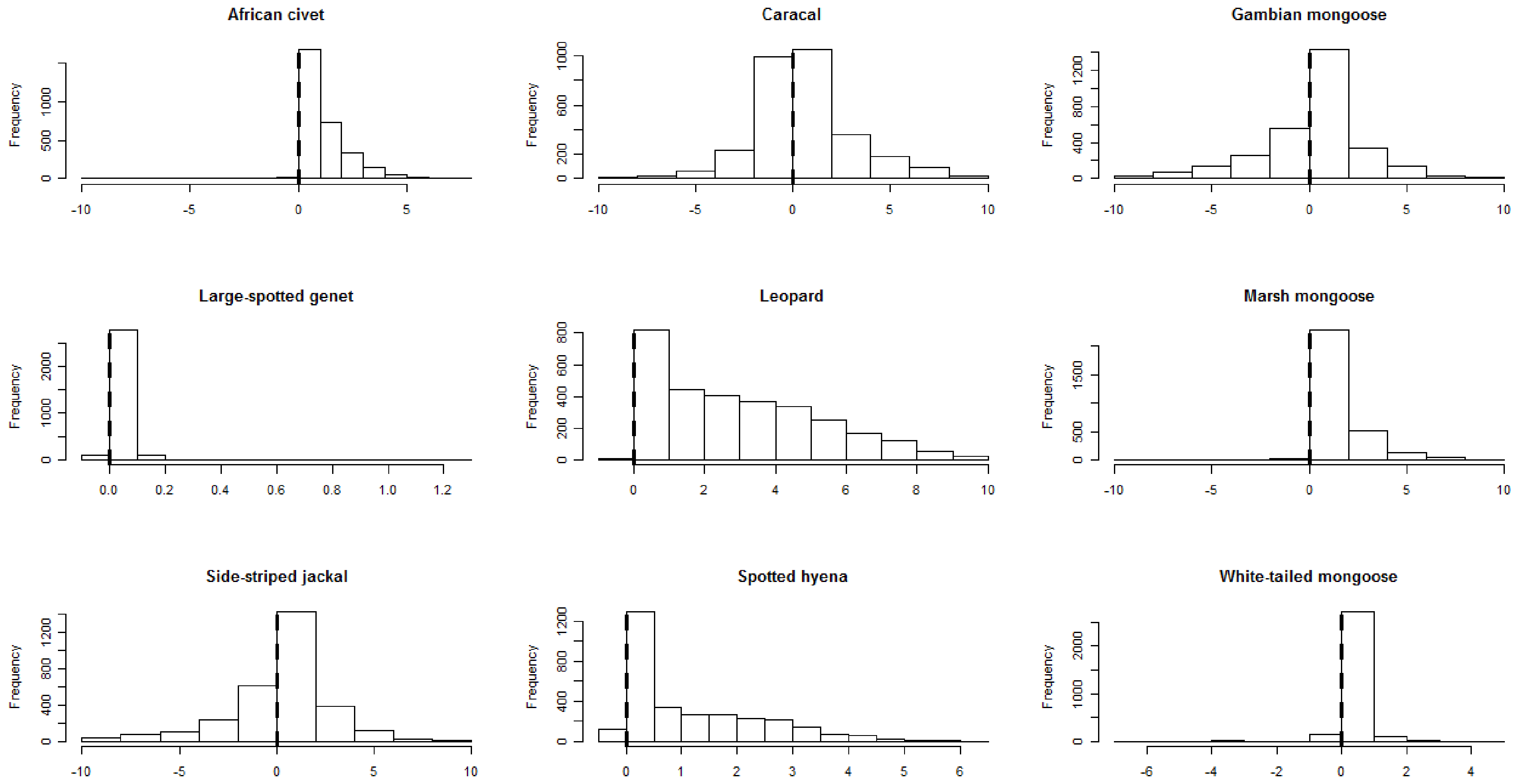

Supplement: Appendix S3 — Posterior distributions for community-level hyperparameters and species-level parameters from the full multi-species occurrence model. (PDF) [file pone.0038007.s005.pdf]
